# Integrative single-cell and spatial transcriptomic analysis reveals that ATG13 enriched in cancer-associated fibroblasts is associated with the suppression of ferroptosis in colorectal cancer

**DOI:** 10.1007/s12672-026-05386-2

**Published:** 2026-06-16

**Authors:** Tong Wang, Jiaming Wu, Peng Wang, Jingjing Huang, Lingfeng Guo, Jialu Guan, Shuo Ning, Jingjing Wang, Wenlu Liu, Fengyu Tian, Zhenghao Luan, Zhongbing Wu, Huayang Pan, Desen Liang, Dawei Wang

**Affiliations:** 1 Key Laboratory of Hepatosplenic Surgery, Ministry of Education, Harbin, China; 2 Department of Anorectal Surgery, The First Affiliated Hospital of Harbin Medical University, Harbin, 150001 China; 3 Department of General Surgery, The Second Affiliated Hospital of Harbin Medical University, Harbin, 150000 China; 4 Binzhou People’s Hospital of Breast Surgery, Shandong, 310053 China; 5 Department of General Surgery, The First Affiliated Hospital of Harbin Medical University, Harbin, 150001 China; 6 WeiFang People’s Hospital, Shandong Second Medical University, Shandong, 261000 China; 7 Department of Gastrosplenic Surgery, The First Affiliated Hospital of Harbin Medical University, Harbin, 150001 China

**Keywords:** Colorectal cancer, Cancer-associated fibroblasts, Single-cell transcriptomics, Spatial transcriptomics, Ferroptosis, ATG13

## Abstract

**Background:**

Colorectal cancer (CRC) progression and metastasis are primarily driven by dynamic changes within the tumor microenvironment (TME). Cancer-associated fibroblasts (CAFs) exhibit significant heterogeneity within the TME and play a crucial role in regulating ferroptosis. Therefore, identifying novel ferroptosis-related biomarkers associated with CAFs could contribute to the development of personalized therapeutic strategies for cancer.

**Methods:**

This study integrates single-cell sequencing data from 64 CRC samples and uses hierarchical clustering to identify cancer-associated fibroblast (CAF) subpopulations. We then combined spatial transcriptomics, hdWGCNA, CellChat, and Monocle analyses to characterize CAF subgroups. Single-cell and spatial transcriptomic data were integrated with Seurat to build a comprehensive spatial atlas. Multiplex immunohistochemical staining was used to analyze the spatial relationships of key genes with CAFs. Functional validation of these genes was performed with molecular docking, qPCR, Western blot, and both cellular and animal experiments.

**Results:**

Single-cell data analysis identified five distinct CAF subpopulations, among which inflammatory cancer-associated fibroblasts (iCAFs) were significantly associated with the ferroptosis pathway and closely correlated with the proliferation and metastasis of malignant epithelial cells (*p* = 0.87). WGCNA revealed that the iCAFs-M10 module exhibited the strongest correlation with the expression of ferroptosis-related genes (cor = 0.6796). Further investigation identified ATG13 as a key hub gene, highly expressed in tumor tissues and significantly associated with poor patient prognosis. Spatial transcriptomics and multiplex immunohistochemistry (mIHC) results demonstrated that ATG13 was highly expressed in CAFs and co-localized with CAFs markers, such as α-SMA and Vimentin. In vitro and in vivo functional assays confirmed that ATG13 knockdown effectively inhibited the proliferation, migration, and invasion of colorectal cancer cells. Additionally, ferroptosis was induced upon ATG13 silencing, as evidenced by increased levels of GPX4, SLC7A11, ACSL4, MDA, Fe^2^⁺, GSH, and lipid peroxidation. In conclusion, this study reveals that ATG13, highly expressed in iCAFs, is associated with ferroptosis suppression in CRC, suggesting a potential CAF-tumor cell crosstalk axis that warrants further mechanistic investigation, thereby promoting the malignant progression of colorectal cancer. These findings provide potential targets for therapeutic strategies aimed at targeting the CAFs-ferroptosis axis.

**Conclusion:**

This study identifies the iCAFs subpopulation as playing a key role in regulating ferroptosis in CRC, with ATG13 acting as a negative regulator of ferroptosis that promotes tumor progression. These findings provide new insights and potential targets for understanding the mechanisms of ferroptosis in CRC and for the development of targeted therapies.

**Supplementary Information:**

The online version contains supplementary material available at 10.1007/s12672-026-05386-2.

## Introduction

Colorectal cancer (CRC) is the third most common malignancy globally. Despite continuous advancements in treatment, its mortality rate remains high, particularly for patients in advanced stages [1–3]. Against this backdrop, the exploration of novel therapeutic targets has become crucial. One such target is ferroptosis, a form of regulated cell death distinct from autophagy and apoptosis. Ferroptosis primarily involves the accumulation of reactive oxygen species (ROS) and the increase in cellular labile iron pool (LIP) levels [4, 5]. The specificity of ferroptosis increases the sensitivity of cancer cells to iron-mediated lipid peroxidation-induced rupture [6–9]. Recently, numerous studies have highlighted that dysregulated iron homeostasis and redox metabolism are common characteristics of cancer. Consequently, a significant alteration in ferroptosis sensitivity has emerged as a promising therapeutic target [10].

Single-cell RNA sequencing (scRNA-seq) enables high-resolution dissection of the tumor ecosystem, thereby revealing cellular heterogeneity. This technology has been widely applied in CRC research, particularly in the study of the TME [11–16]. It plays a crucial role in advancing our understanding of cell subtypes and their interactions within the TME. However, a limitation of single-cell RNA sequencing is its inability to provide spatial information, which restricts comprehensive studies on the dynamic molecular trajectories during CRC tumorigenesis. Spatial transcriptomics, however, addresses this gap [17–19]. Spatial transcriptomics preserves the spatial context of RNA, facilitating the exploration of the biological significance of tumor location differences [20, 21]. Therefore, our goal is to integrate these two technologies to gain a comprehensive understanding of cellular alterations and their functions in CRC, thereby contributing to the discovery of novel therapeutic targets.

CAFs play a crucial role in cancer initiation, progression, and treatment response [22]. As key components of the tumor microenvironment, CAFs exhibit highly diverse functions. They not only support cancer cell survival by secreting various cytokines, chemokines, and growth factors, but also enhance cancer cell invasiveness by activating the epithelial-mesenchymal transition (EMT) pathway or by promoting malignancy by stimulating tumor stem cell properties [23]. CAFs are derived from various sources and exhibit significant phenotypic and functional plasticity in their interactions with tumor cells. Studies utilizing scRNA-seq have identified distinct CAFs subpopulations with unique gene expression profiles and functional characteristics. These subpopulations, including myofibroblastic CAFs (myCAFs), inflammatory CAFs (iCAFs), and antigen-presenting CAFs (apCAFs), have been shown to influence tumor progression and immune response through different mechanisms [24–28]. The application of scRNA-seq has further revealed the complexity of CAFs, including their heterogeneity and plasticity. However, most studies rely on a single histological feature to analyze tumors and do not integrate scRNA-seq with spatial transcriptomics or CAFs. Therefore, we aim to leverage the strengths of all three approaches to provide new opportunities for the development of novel CAFs-targeted therapeutic strategies.

This study aims to integrate single-cell and spatial omics technologies to uncover the regulatory mechanisms of ferroptosis in CRC CAF subpopulations. We used scRNA-seq data to fine-cluster cell types within the CRC microenvironment and combined this with spatial transcriptomic data to reveal the spatial locations and interactions of cell subpopulations, with a focus on CAF subtypes and their interactions with tumor cells. We further explored the regulatory relationship between core CAFs subpopulations and ferroptosis and identified CAFs+ ferroptosis-related genes. These genes were found to have high accuracy in promoting CRC progression, offering new theoretical insights and potential targets for CRC diagnosis and treatment.

## Methods

### Data collection and process

scRNA-seq data (GSE166555, GSE146771, GSE132465, GSE144735, GSE188711) were retrieved from the GEO database (https://www.ncbi.nlm.nih.gov/geo/), and the scRNA-seq data from 57 CRC samples were downloaded. Additionally, scRNA-seq data (EMTAB8107) were obtained from the ArrayExpress database (https://www.ebi.ac.uk/), with scRNA-seq data from 7 CRC samples downloaded.

The scRNA-seq data were processed using Seurat (version V4.1.1). Cells with mitochondrial content greater than 20%, erythrocyte content exceeding 1%, fewer than 300 expressed genes, or more than 5,000 expressed genes, as well as the top 3% of cells with the highest RNA counts, were excluded from the analysis. Data normalization, cell clustering, and dimensionality reduction were performed using the Seurat package. Gene expression data were normalized by multiplying gene scores by 10,000 and converting to natural logarithms. The top 2,000 most variable genes were selected for scaling, and gene expression data were normalized using the ScaleData function. Principal component analysis (PCA) was then performed using the “RunPCA” function. Batch correction was applied using the "HarmonyIntegration" function from the Harmony R package. A cell adjacency graph was constructed using the FindNeighbors function (dims = 1:30), followed by cell clustering with the FindClusters function (resolution = 0.6), yielding a final dataset of 160,571 cells. These cells were projected into low-dimensional space using t-SNE. Non-linear dimensionality reduction was performed using the RunUMAP function, and manual cell annotation of subpopulations was conducted based on marker genes. All downstream correlation analyses were performed on Harmony-corrected data to minimize inter-sample batch effects. Cell subpopulation scores were computed using the AddModuleScore function in Seurat, which calculates per-cell enrichment scores for predefined gene sets relative to randomly sampled control genes. A curated ferroptosis-related gene set was retrieved from the FerrDb database (http://www.zhounan.org/ferrdb), a comprehensive repository of experimentally validated ferroptosis regulators, drivers, and suppressors.

### Identification of malignant epithelial cell

To identify malignant epithelial cells, the inferCNV package in R was used to distinguish between malignant and non-malignant epithelial cells. The settings for inferCNV analysis included a cutoff of 0.1, with clustering by group set to TRUE and activation of the Hidden Markov Model for CNV estimation. Lymphocyte and myeloid cell clusters were used as reference cell populations, with a threshold of 0.5 applied to reduce false positives in copy CNV inference. Epithelial cells (ECs) with lower CNV scores that clustered with reference cells were classified as non-malignant, while ECs with higher CNV scores were designated as malignant.

### Construction of weighted gene co-expression networks

To identify hub genes significantly associated with iCAFs, the 'hdWGCNA' package was used, which is specifically designed to address the sparsity and non-independence inherent in single-cell RNA sequencing data. Prior to network construction, genes expressed in at least 5% of cells (fraction = 0.05) were retained as input to reduce the impact of dropout-driven sparsity. To further mitigate single-cell sparsity and cell-to-cell dependency, metacells were constructed using the MetacellsByGroups function (k = 25 nearest neighbors, max_shared = 10, reduction = 'harmony'), which aggregates transcriptionally similar cells into representative metacell profiles based on Harmony-corrected embeddings. This metacell aggregation strategy is analogous to pseudo-bulk approaches and has been demonstrated to improve the reliability of co-expression network inference from single-cell data. The iCAFs subpopulation was specifically selected for SetDatExpr analysis using normalized RNA assay data. A signed co-expression network was constructed using a soft-thresholding power of 8, which was selected based on the scale-free topology criterion (R^2^ > 0.80) as assessed by TestSoftPowers. Hierarchical clustering of the resulting topological overlap matrix (TOM) was used to identify ten co-expression modules (M1–M10), with grey module genes excluded from downstream analysis.

### CellChat analysis and cell trajectory analysis

Cell–cell communication with biological significance was predicted and visualized using the 'CellChat' package to assess intercellular communication for each specific signaling pathway. Pseudotime trajectory analysis was performed using the 'Monocle' package to map the differentiation processes of different cell subtypes. The settings for the analysis included reduction_method = "DDRTree" and max_components = 3. Cells were ordered according to their pseudotime using the 'orderCells()' function. Visualization of the results was performed using the 'plot_genes_branched_heatmap' function.

### Gene set enrichment analysis

We used the R package GSVA (version 1.46.0) to analyze the gene set enrichment of 50 hallmark pathways from the MsigDB.

### Survival analysis

Survival data from TCGA-CRC (https://www.cancer.gov/) patients were used, with samples lacking survival information excluded, resulting in a total of 547 CRC samples with available survival data. Based on gene expression values, the patients were divided into high-expression and low-expression groups using the surv_cutpoint() function. Kaplan–Meier (KM) survival analysis was performed to assess overall survival (OS) between the high-expression and low-expression groups, and comparisons were made using a two-sided log-rank test.

### Spatial proteomic analysis

Spatial transcriptomic data were processed in Seurat. The Spatial assay was normalized using NormalizeData with the LogNormalize method and a scale factor of 10,000. A CAF gene signature was defined based on canonical fibroblast/CAF markers, including FAP, PDPN, PDGFRA, THY1, COL1A1, COL1A2, COL3A1, COL6A1, COL6A2, DCN, LUM, POSTN, TNC, CXCL12, MMP2, and CTHRC1. A per-spot CAF signature score was then calculated using Seurat AddModuleScore.ATG13 expression values and spatial coordinates were extracted for each spot and merged into a unified data table. Spot-level association between the CAF score and ATG13 expression was assessed using Spearman correlation. To further quantify local spatial association, a k-nearest-neighbor approach was applied using spot coordinates (x, y) with k = 6. For each spot, the mean CAF score and mean ATG13 expression across its 6 nearest neighboring spots were calculated to generate spatially smoothed CAF and ATG13 values. Pearson correlation analysis was then performed between the smoothed CAF score and smoothed ATG13 expression (Fig. S4).

### Molecular docking

First, the PDB files for ATG13 (PDB Entry-5xv4) and CREB1 (PDB Entry-5zk1) were downloaded from the RCSB PDB database (https://www.rcsb.org/pdb/home/home.do) for protein docking studies. Subsequently, Discovery Studio 4.1 software was downloaded from the official website. The PDB files for ATG13 and CREB1 were then imported into the software, and the amino acid binding sites of both proteins in their spatial structures were analyzed.

### Western blotting

Protein samples extracted from tissues or cells were separated by SDS-PAGE electrophoresis and transferred to a PVDF membrane. The membrane was blocked with 5% skim milk at room temperature for 1 h. The PVDF membrane was then incubated with the primary antibody at 4 °C overnight. Subsequently, the membrane was incubated with the secondary antibody at room temperature for 1 h, and the immunoblot was visualized using an ECL chemiluminescent substrate.

### Quantitative real-time PCR

Total mRNA was extracted using the AxyPrep Total RNA Extraction Kit. mRNA was then reverse transcribed using the TaKaRa reverse transcription kit. Quantitative PCR (qPCR) was performed using the ABI 7300 QuantStudio 3 PCR system with ChamQ SYBR qPCR Master Mix (Q341-02; Vazyme) for detection.

### Cell function assays

HCT116 cells transfected with si-NC or si-ATG13 (1 × 10^3^ cells/mL) were seeded in a 96-well plate. The cells were cultured at 37 °C for 4 days, as per the experimental requirements. Subsequently, 10 µL of CCK-8 solution (Seven, Beijing, China) was added to each well, and the cells were incubated at 37 °C for an additional 2–3 h. The absorbance of each well was then measured at 450 nm using a microplate reader.

For the colony formation assay, cells were seeded at a density of 500 cells per well in a 6-well plate, and the medium was changed every 3 days. After approximately 2 weeks, the cells were fixed with 4% paraformaldehyde at room temperature for 30 min, followed by staining with 0.1% crystal violet for 30 min.

Migration and invasion assays were conducted using 24-well transwell plates with 0.8 µm pore size. A total of 3 × 10^4^ cells were suspended in the upper chamber, either with or without matrigel, and cultured in serum-free medium. The lower chamber contained medium supplemented with 20% fetal bovine serum (FBS). After 24 h of incubation, the cells in the upper chamber were stained with crystal violet and observed under a microscope.

Cells were seeded at a density of 10,000 cells per well in a 96-well plate. The following day, cells were incubated with EdU solution (1:1000) for 2 h. The cells were then fixed with 4% paraformaldehyde for 20 min, followed by incubation with a commercial kit for 30 min. Subsequently, the cells were washed with 0.5% Triton X-100 for 10 min. Finally, the cells were incubated with DAPI solution at room temperature for 10 min."

### Immunofluorescence (IF) assay and immunohistochemical staining

First, the tissue sections were deparaffinized and rehydrated using xylene and ethanol. The sections were then subjected to antigen retrieval by boiling in citrate buffer for 10 min. Afterward, the sections were incubated with 3% hydrogen peroxide (H₂O₂) at room temperature for 10 min. Following blocking with goat serum, the sections were incubated overnight at 4 °C with the primary antibody. The next day, the sections were washed three times with PBST at room temperature and then incubated with the secondary antibody at room temperature for 30 min. Finally, the sections were stained with DAB and hematoxylin, and images were captured using an N2-Mi8 microscope.

Tumor tissues were fixed with 4% paraformaldehyde, embedded in paraffin, rehydrated, and subjected to antigen retrieval. The sections were incubated with primary antibodies against ATG13, α-SMA, and Vimentin, followed by incubation with goat anti-mouse IgG H&L and goat anti-rabbit secondary antibodies. Images were captured using a fluorescence microscope (Olympus)."

### Transmission electron microscopy (TEM)

After preprocessing, the cells were collected and pelleted, then fixed with 2.5% glutaraldehyde solution at 4 °C. Transmission electron microscopy imaging was performed using a SEM (scanning electron microscope).

### Fe^2+^, BODIPY C11, MDA, ROS, GSH assay

Ferroptosis indicators were measured using a range of assays, including the ferrous iron assay kit, lipid peroxidation (BODIPY C11) assay kit, MDA assay kit, reactive oxygen species (ROS) assay kit, and reduced glutathione (GSH) assay kit.

### Animal model

Six 4–6-week-old BALB/c nude mice were housed at the Harbin Medical University Laboratory Animal Center and used to establish in vivo tumor models. The mice were randomly divided into experimental and control groups, with 3 mice per group. For subcutaneous cell line xenografts, 2 × 10^6^ HCT116 cells were suspended in 100 µL of PBS and injected subcutaneously into the mice. At the end of the experiment, all mice were euthanized by isoflurane anesthesia, and tumor tissues were harvested for measurement analysis. All experimental procedures were conducted in accordance with the guidelines for animal care established by the U.S. All animal experiments were approved by the Institutional Animal Ethics Committee of Harbin Medical University. Tumor volume was calculated as V = 0.5 × (length × width2). The maximal tumor size permitted by the institutional ethics committee was 2,000 mm^3^. In all animal experiments, this limit was not exceeded.

### Statistical analysis

All data were subjected to statistical analysis using GraphPad Prism software (GraphPad Prism 8). Experimental data are presented as the mean ± standard deviation (SD). Intergroup differences were analyzed using t-tests, one-way or two-way analysis of variance (ANOVA). A p-value of < 0.05 was considered statistically significant. For Spearman correlation analyses conducted at the single-cell level, we note that p-values may be inflated due to the large number of cells analyzed; therefore, the correlation coefficient (rho) is reported as the primary measure of effect size. To validate key correlations, pseudo-bulk analyses were additionally performed by averaging module scores per patient sample prior to correlation calculation.

## Results

### The single-cell transcriptomic analysis of the CRC cell atlas

**Fig. 1:**
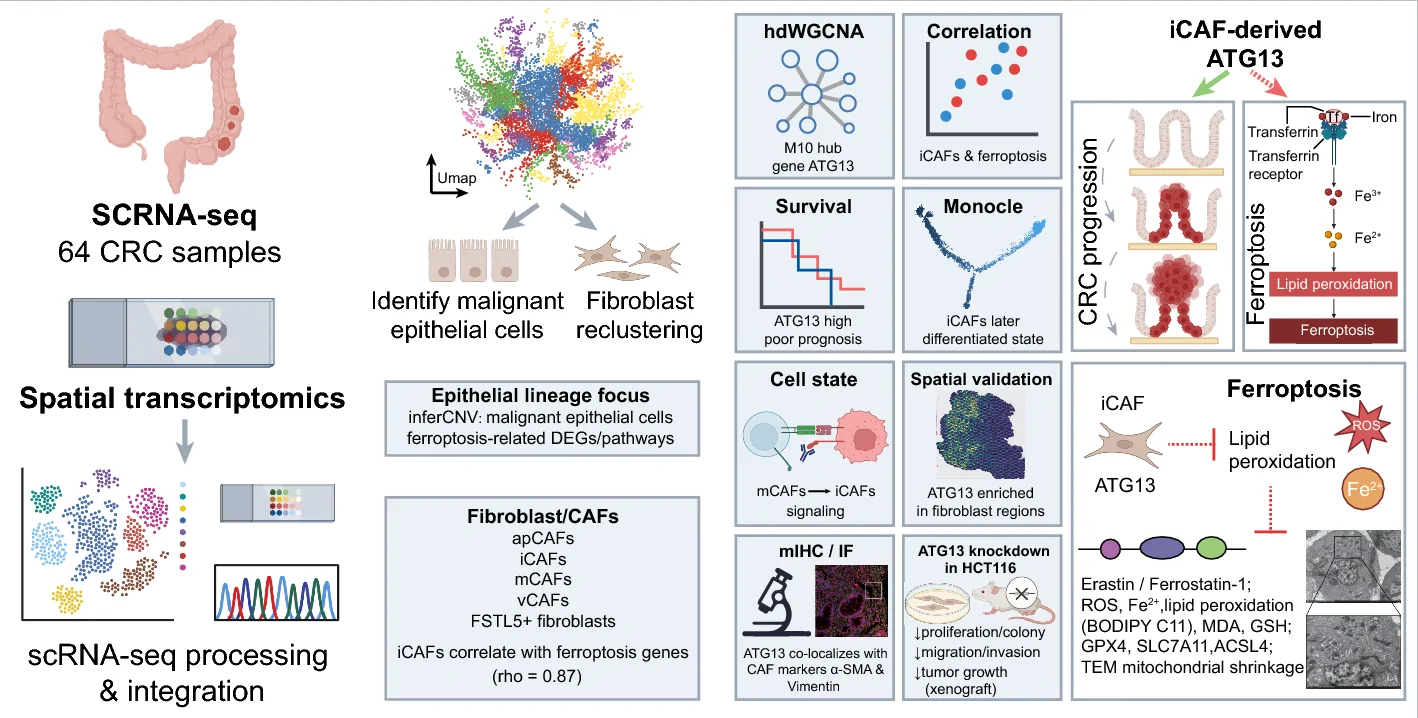
Study workflow schematic

**Fig. 2:**
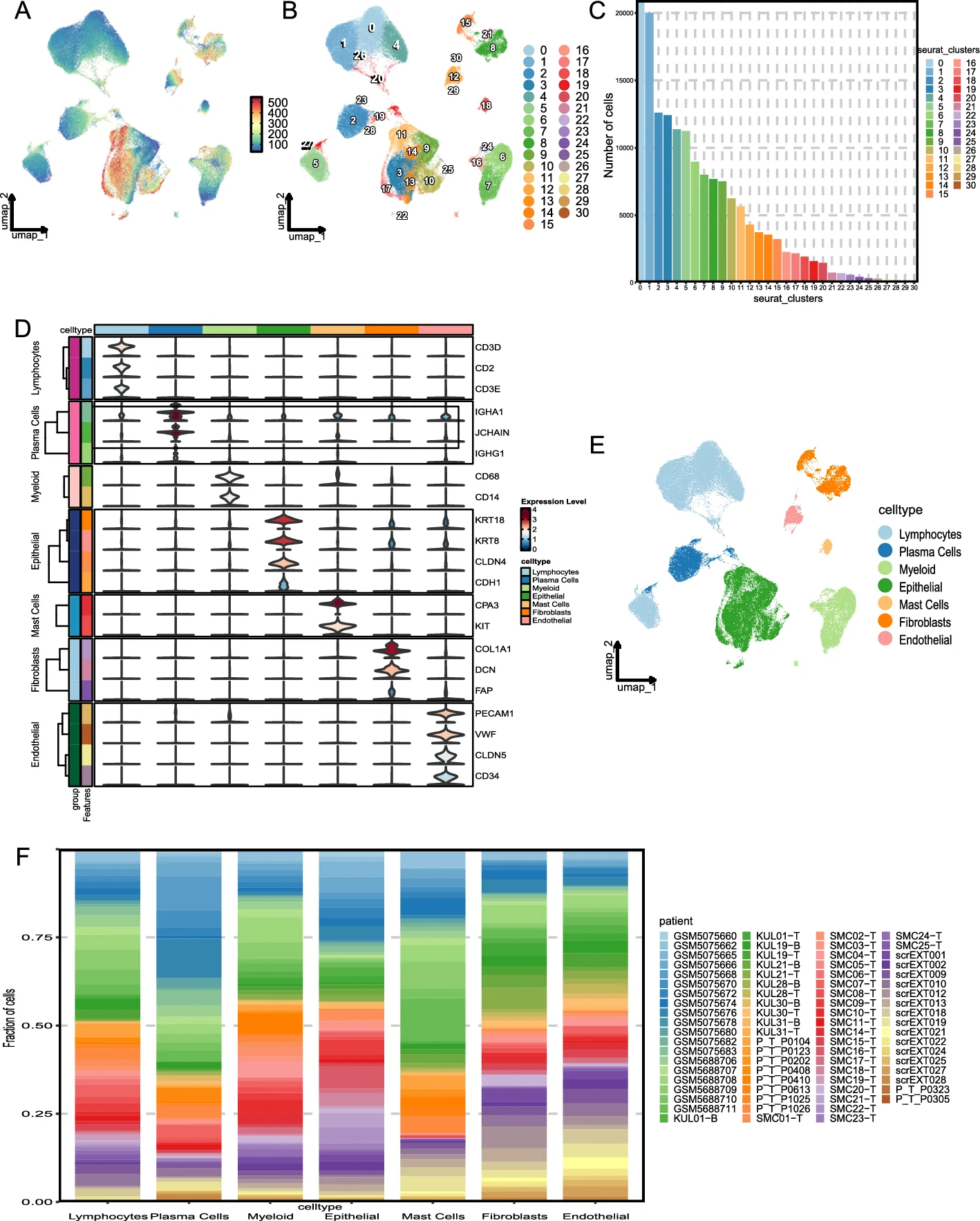
The single-cell transcriptomic analysis of the CRC cell atlas. **A** UMAP reveals cellular origin. **B** Cellular annotation results. **C** Cell count for each cluster after annotation. **D** Marker gene expression. **E** UMAP reveals cellular classification. **F** Proportions of patients in each cell type

Figure 1 illustrates the analytical process workflow. To elucidate potential mechanisms underlying CRC development, we analyzed single-cell transcriptomic data from 64 CRC samples obtained from public databases. After standard preprocessing and quality control of the single-cell data, we constructed a CRC single-cell atlas comprising 160,571 single cells (Fig. 2A). t-SNE clustering analysis of the single-cell data revealed 31 cell clusters, which were annotated into 7 cell types based on specific marker genes: lymphocytes (65,543 cells), plasma cells (14,962 cells), myeloid cells (19,655 cells), epithelial cells (42,301 cells), mast cells (1,923 cells), fibroblasts (11,625 cells), and endothelial cells (4,562 cells) (Fig. 2B–E). To explore the cellular heterogeneity, we further compared the infiltration proportions of the 7 cell types in CRC tissue, with epithelial cells showing a significantly higher infiltration ratio (Fig. 2F).

### Single-cell transcriptomics reveals the potential role of malignant epithelial cells in tumor progression

**Fig. 3:**
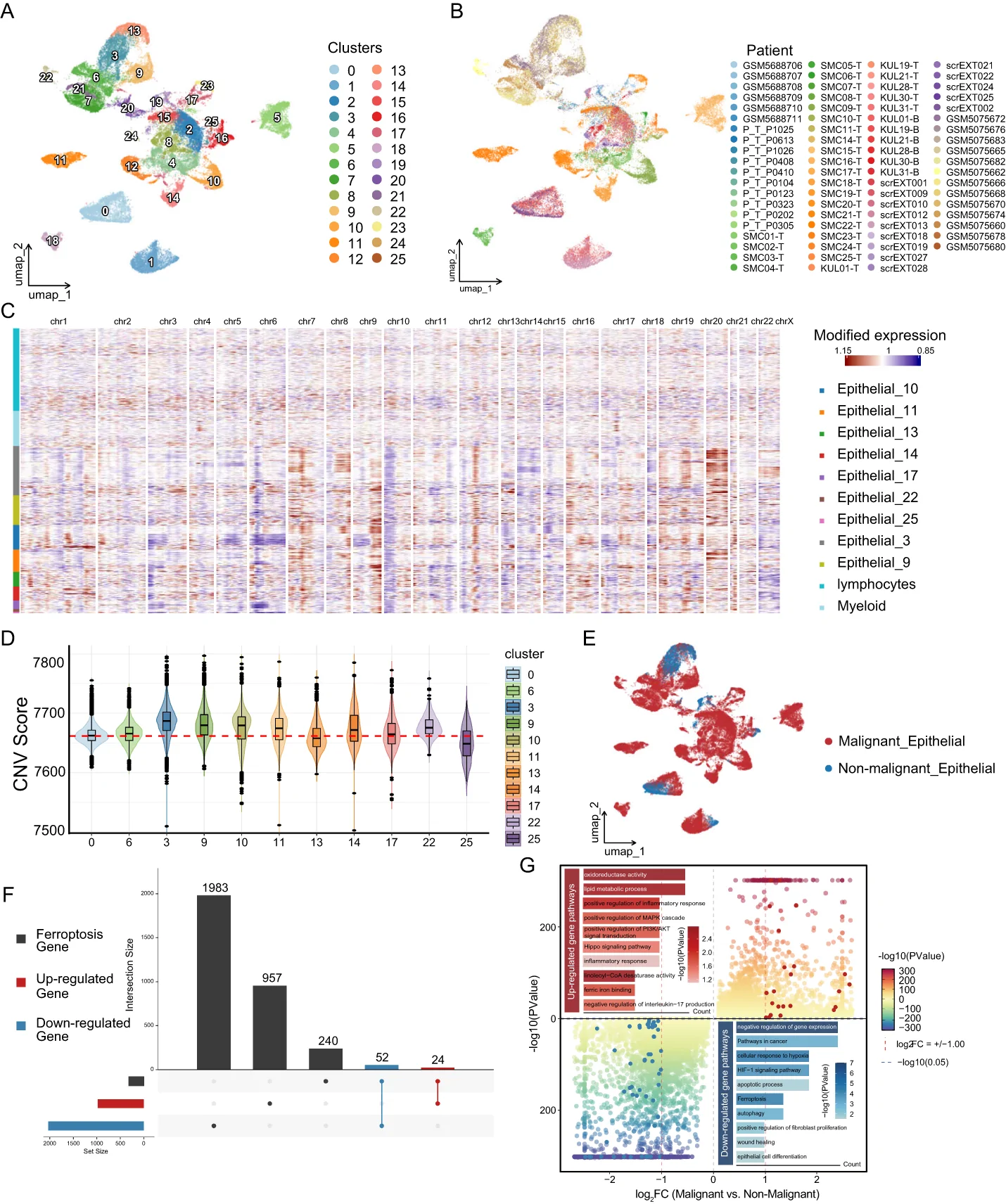
Single-cell transcriptomics reveals the potential role of malignant epithelial cells in tumor progression. **A** Cellular annotation results. **B** UMAP plot of epithelial cells based on patient classification. **C** Heatmap of copy number variation (CNV) in epithelial cells. **D** Boxplot of CNV scores. **E** UMAP plot distinguishing malignant from non-malignant epithelial cells. **F** Intersection analysis of ferroptosis-related genes with upregulated and downregulated genes. **G** Differential gene enrichment analysis

Given the critical role of epithelial cells in colorectal cancer (CRC) cell origin [29], we performed single-cell sequencing analysis. Using t-SNE dimensionality reduction, we reclustered epithelial cells into 26 groups (Fig. 3A, B). The R package inferCNV was used to identify malignant epithelial cells by analyzing chromosomal copy CNV and assigning CNV scores. Lymphocyte and myeloid cell clusters served as normal reference cells to further infer CNV profiles in epithelial cells (Fig. 3C, D). Hierarchical clustering of these profiles revealed 36,076 malignant epithelial cells (Fig. 3E). To identify ferroptosis-related differentially expressed genes, the complete set of upregulated and downregulated DEGs (filtered by |log2FC|≥ 0.1, minimum expression in ≥ 1% of cells, Wilcoxon rank-sum test) were independently intersected with the FerrDb-derived ferroptosis gene set. Genes present in both the DEG list and the ferroptosis gene set were designated as ferroptosis-related DEGs and subjected to enrichment analysis. Malignant epithelial cells, which require substantial antioxidant consumption during proliferation, were further investigated for their relationship with ferroptosis. To explore this, the intersection of the differentially expressed genes with a ferroptosis gene set identified 24 upregulated ferroptosis-related genes (FERGs) and 52 downregulated FERGs (Fig. 3F). Enrichment analysis then showed that these 76 ferroptosis-related differentially expressed genes (FDEGs) were primarily associated with ferroptosis, the PI3K-Akt signaling pathway, and the MAPK signaling pathway. Other enriched pathways included oxidoreductase activity, lipid metabolism processes, and HIF-1 signaling (Fig. 3G). Notably, CAFs are key components of the tumor microenvironment in solid tumors and play a pivotal role in regulating the behavior of malignant epithelial cells (EPCs) [30]. Subsequent dimensionality reduction and visualization revealed that FDEGs expression was elevated in epithelial cells, fibroblasts, and myeloid cells.

### Single-cell transcriptomics reveals the molecular heterogeneity and functional diversity of CAFs

**Fig. 4:**
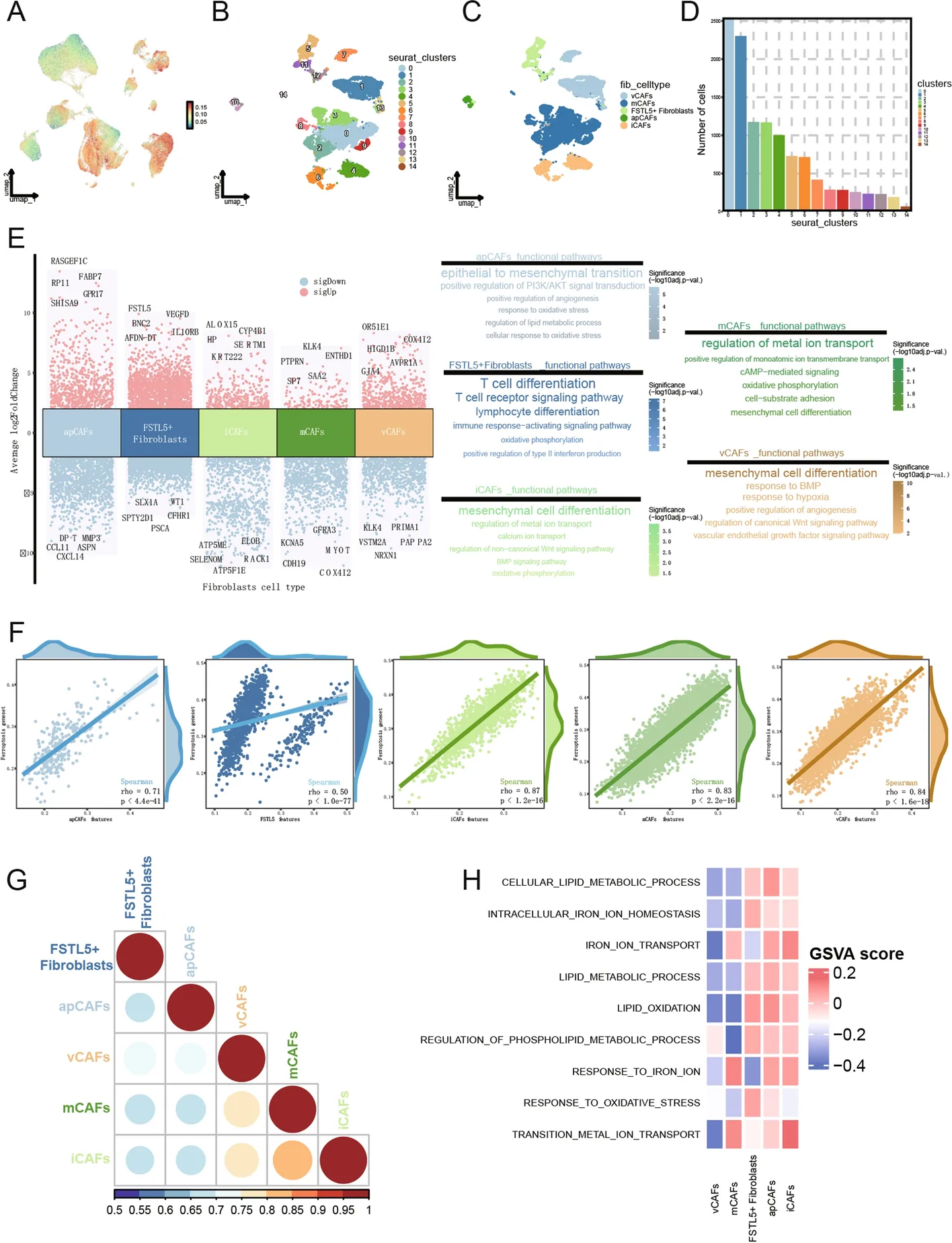
Single-cell transcriptomics reveals the molecular heterogeneity and functional diversity of CAFs. **A** UMAP reveals ferroptosis gene expression scoring. **B** UMAP reveals fibroblast origin. **C** Fibroblast annotation results. **D** Cell count for each cluster after annotation. **E** Differential enrichment analysis of fibroblasts. **F** Correlation between fibroblast subpopulations and ferroptosis. **G** Correlation among fibroblast subpopulations. **H** GSVA-based enrichment of ferroptosis-related pathways in fibroblast subpopulations

Through AddModuleScore analysis, the ferroptosis-related gene scores for each cell type in the CRC tumor microenvironment were calculated. This revealed high expression of ferroptosis-related genes in both the epithelial and fibroblast subpopulations (Fig. 4A). After reclustering and annotating the fibroblast cells, five distinct cell types were identified based on marker gene expression. These include: antigen-presenting fibroblasts (apCAFs, 257 cells), FSTL5-positive fibroblasts (FSTL5 + Fibroblasts, 1,196 cells), inflammatory fibroblasts (iCAFs, 1,720 cells), matrix fibroblasts (mCAFs, 5,536 cells), and vascular-associated fibroblasts (vCAFs, 2,916 cells) (Fig. 4B–D). Differentially expressed genes (DEGs) were identified in these subpopulations to elucidate the distinct roles of the five fibroblast subtypes. KEGG enrichment analysis showed that apCAFs were significantly enriched in biological processes such as epithelial-mesenchymal transition (EMT), lipid metabolism, and oxidative stress (Fig. 4E). FSTL5+ Fibroblasts exhibited increased activity in immune-related functions, including T-cell differentiation, lymphocyte differentiation, and positive regulation of type II interferon production. This may assist the tumor in immune evasion (Fig. 4E). Moreover, mCAFs showed enrichment in biological processes related to cell–matrix adhesion and mesenchymal cell differentiation. This suggests their association with poor prognosis and active participation in tumor invasion and migration (Fig. 4E). vCAFs exhibited heightened activity in angiogenesis and vascular endothelial growth factor signaling pathways (Fig. 4E). Notably, iCAFs were not only involved in the regulation of the non-canonical Wnt signaling pathway but also in the modulation of mesenchymal cell differentiation and metal ion transport (Fig. 4E). Additionally, Spearman correlation analysis at the single-cell level revealed a strong positive correlation between iCAFs module scores and ferroptosis-related gene set scores (rho = 0.87), the highest among all five CAFs subpopulations (Fig. 4F). This association was further confirmed at the patient-level pseudo-bulk analysis (Spearman’s rho = 0.986, *p* = 2.71e−30), supporting the robustness of this finding independent of cell number-driven statistical inflation (Fig. S1). This result suggests that the iCAFs subpopulation may play a pivotal role in regulating ferroptosis in the tumor microenvironment. Figure 4G demonstrates a significant correlation between iCAFs, mCAFs, and vCAFs subpopulations. GSVA analysis further revealed that iCAFs showed higher enrichment in ferroptosis-related pathways, including intracellular lipid metabolism, iron ion transport, and lipid oxidation, compared with other fibroblast subpopulations (Fig. 4H). These findings suggest that iCAFs may be associated with ferroptosis in cells and contribute to the proliferation and metastasis of colorectal cancer cells by modulating malignant epithelial cells, correlating with poor prognosis in patients.

### iCAF-enriched ATG13 is associated with ferroptosis regulation and poor prognosis in CRC

**Fig. 5:**
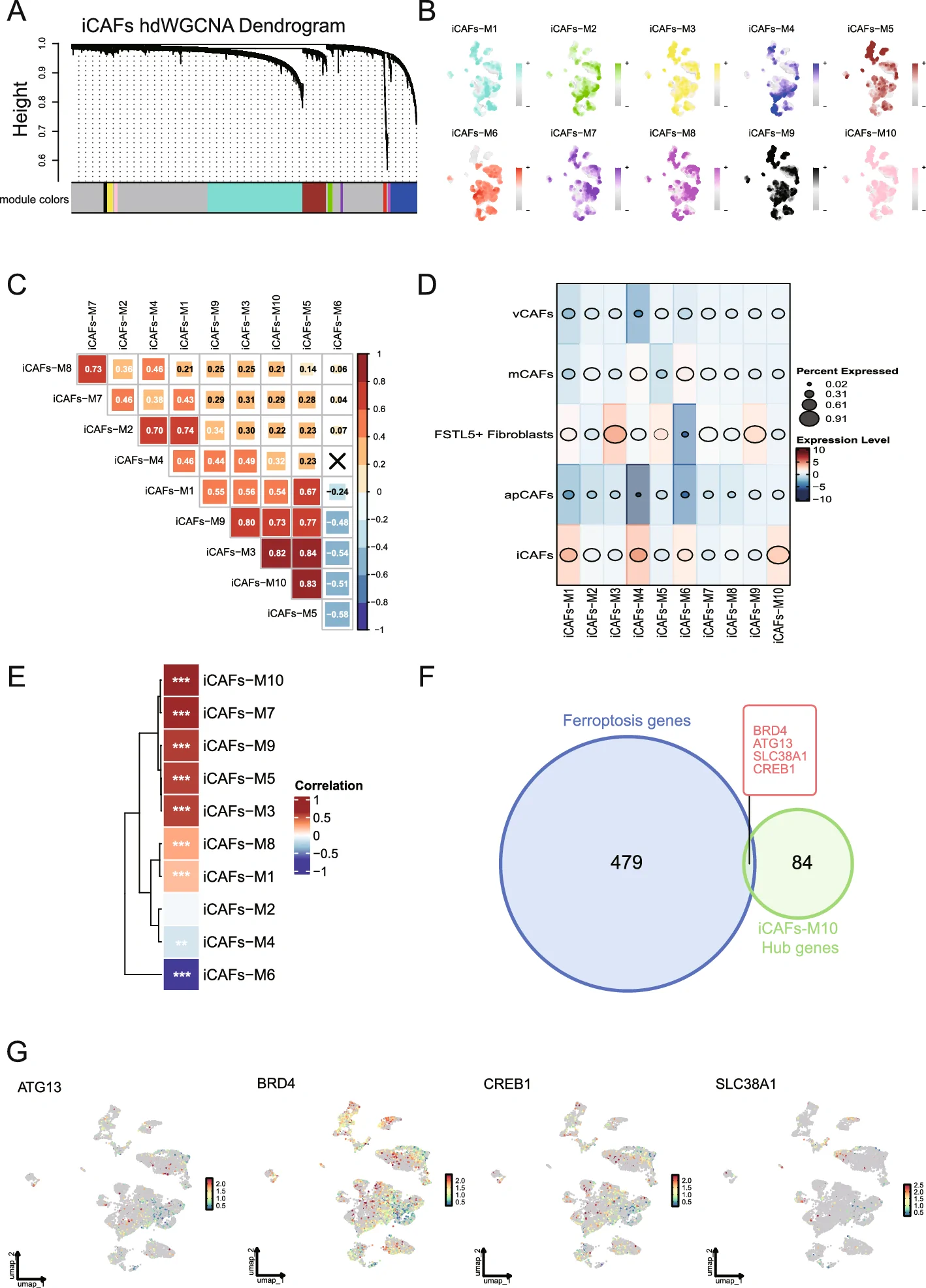
iCAFs-enriched ATG13 participates in the ferroptosis process and impacts CRC prognosis. **A** Clustering map of genes in iCAFs fibroblasts, divided into 10 modules. **B** Gene expression score mapping of modules 1–10 at single-cell resolution. **C** Correlation between the 10 modules identified in Fib-iCAFs. **D** Expression of Fib-iCAFs module genes across fibroblast subpopulations. **E** Correlation between Hub genes of Fib-iCAFs modules and ferroptosis gene expression. **F** Intersection of Hub genes in the iCAFs-M10 module with ferroptosis genes, yielding 4 genes. **G** UMAP reveals the expression distribution of the 4 genes in the Fib subpopulation

To explore the gene co-expression patterns in the iCAFs subpopulation, we applied the hdWGCNA framework, which is specifically optimized for single-cell data through metacell aggregation (k = 25) to address transcriptomic sparsity. Ten gene co-expression modules (M1–M10) were identified from the resulting metacell-level co-expression network (Fig. 5A). Among them, expression of Hub genes in the M10 module had the strongest correlation with ferroptosis-related genes (r = 0.6796, *p* < 1.083503e−52). This suggests the module may be involved in regulatory processes related to ferroptosis (Fig. 5B–E). Further intersection analysis with the ferroptosis gene set in iCAFs-M10 revealed four ferroptosis-related Hub genes: BRD4, ATG13, SLC38A1, and CREB1 (Fig. 5F). After checking Hub gene expression in the GEPIA database and analyzing survival for 547 CRC samples with available survival information (TCGA), we found that only high expression of ATG13 in CRC tumor tissue was significantly associated with poor prognosis (Fig. S2). Transcription factor network analysis of iCAFs-M10 predicted CREB1 as a potential transcription factor involved in the regulation of ATG13 (Fig. S3). In conclusion, iCAF-enriched ATG13, is highly expressed in CRC tumor tissue and correlates with poor prognosis in CRC patients.

### Single-cell analysis reveals the developmental trajectories of CAFs subpopulations

**Fig. 6:**
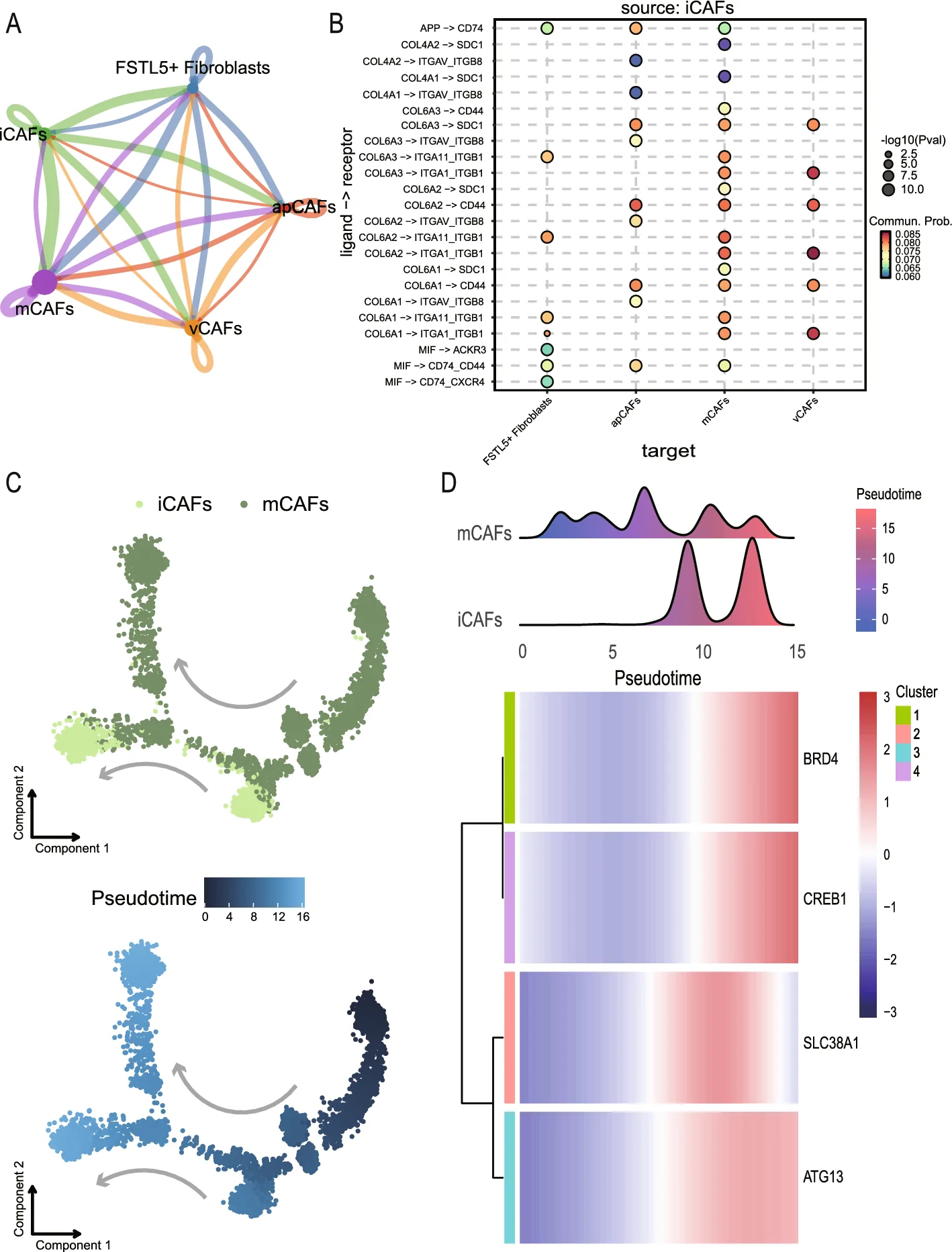
Single-cell analysis reveals the developmental trajectories of CAFs subpopulations. **A** Cell interaction network diagram among fibroblast subpopulations. **B** Ligand bubble plot of Fib-iCAFs versus other subpopulations. **C** Pseudotemporal analysis of iCAFs and mCAFs subpopulations. **D** Temporal variation of the 4 genes in pseudotemporal analysis

To explore the cellular communication network within CAF subpopulations, we used CellChat to investigate interactions between CAF subgroups. The cell interaction network revealed significant communication between iCAFs and other fibroblast subpopulations (Fig. 6A). Notably, mCAFs act as signal senders, secreting specific ligands that bind to receptors on iCAFs, thereby regulating their functions (Fig. 6B). Pseudotime trajectory analysis showed a distinct branching structure, suggesting that iCAFs and mCAFs may follow different developmental paths. Figure 6C demonstrates that mCAFs might represent an earlier stage, whereas iCAFs appear to be a more mature differentiated state. Heatmap analysis showed that BRD4, ATG13, SLC38A1, and CREB1 are highly expressed during the later stages of differentiation (Fig. 6D). Notably, during the differentiation process of iCAFs and mCAFs, the high expression of BRD4 and ATG13 may indicate that the cells are in a more extreme state, potentially due to stress experienced during differentiation. This stress could induce oxidative stress, leading to iron accumulation and activation of the ferroptosis pathway, thereby impacting cell survival and function, and influencing patient prognosis and disease progression.

### ATG13 and CAFs co-localize spatially

**Fig. 7:**
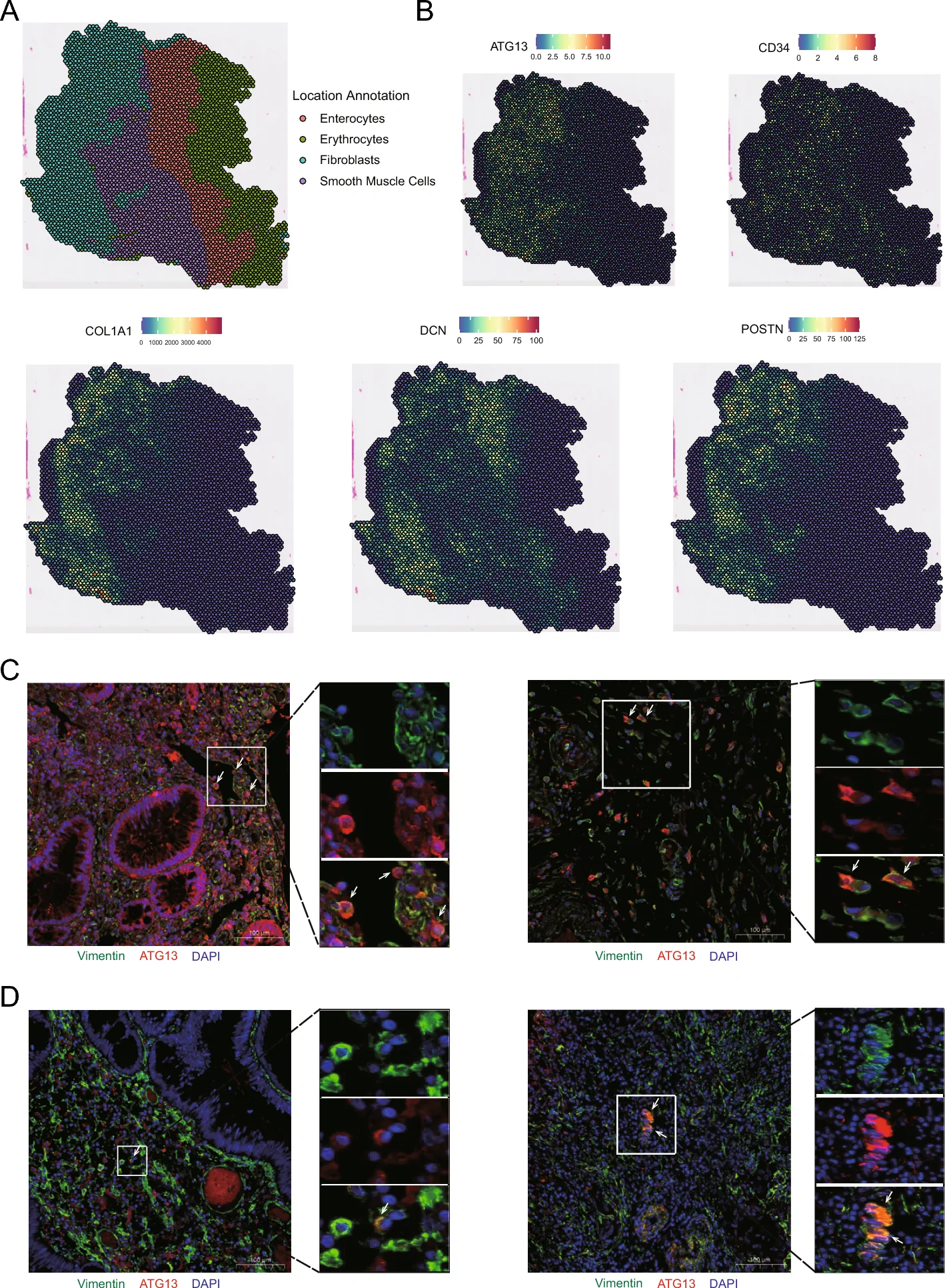
ATG13 and CAFs co-localize spatially. **A** Spatial distribution of different cell types in CRC tumor tissue sections. **B** Expression of CAFs marker genes: iCAFs markers DCN, COL1A1; mCAFs marker POSTN; ATG13. **C**, **D** Multiplex immunofluorescence demonstrating tissue samples from CRC tumor tissues, with green indicating α-SMA and vimentin positivity; red indicating ATG13 positivity; blue representing DAPI staining for nuclear identification. Scale bar: 100 µm

Furthermore, we integrated single-cell RNA sequencing data with spatial transcriptomics using Seurat software, thereby reconstructing a spatial single-cell atlas. We observed high ATG13 expression in the fibroblast region of CRC tissues, with levels similar to those in areas where mCAFs marker genes were highly expressed (Fig. 7A, B). Additionally, tissue immunofluorescence analysis confirmed the co-localization of ATG13 with CAFs markers α-SMA and Vimentin (Figs. 7C, D, S5A,B). Overall, these findings suggest that high ATG13 expression in iCAFs plays a crucial role in remodeling the tumor microenvironment and may promote the malignant progression and invasion of CRC through interactions with mCAFs, thereby contributing to a poor prognosis.

### ATG13 promotes CRC cell proliferation, migration, and invasion, and is spatially enriched in iCAF regions

**Fig. 8:**
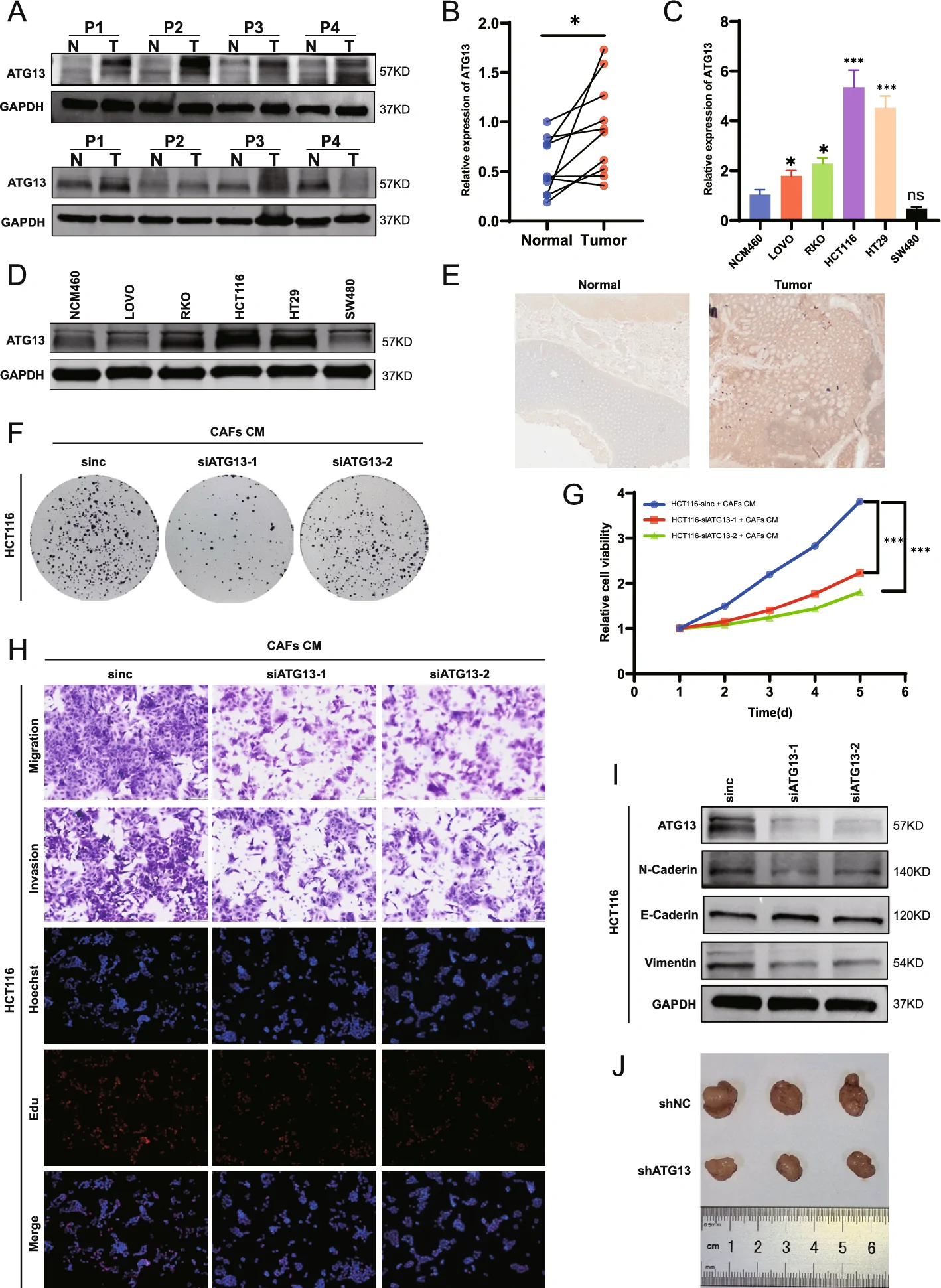
iCAFs-enriched ATG13 promotes CRC cell proliferation, migration, and invasion. **A** Western blot analysis of ATG13 expression in CRC tissues and matched adjacent non-tumor tissues. **B** mRNA levels of ATG13 in CRC tissues and adjacent non-tumor tissues. **C** qRT-PCR analysis of ATG13 expression in CRC cell lines. **D** ATG13 protein levels and relative band density in CRC cell lines. **E** Representative images of ATG13 immunohistochemical staining in normal intestinal epithelium and colorectal cancer tissues. **F** Representative images of colony formation and statistical analysis of colony count. Scale bar: ×400, 50 µm. **G** CRC cell growth curve analysis based on CCK-8 assay. **H** Representative images of transwell migration, Matrigel invasion assays, and EDU assay for the indicated cells. Scale bar: 100 µm. **I** Western blot reveals changes in malignant biomarker protein expression in CRC cells transfected with specific plasmids. **J** Representative images of subcutaneous xenograft tumors from CRC cells

We examined ATG13 expression in CRC tumor tissues and adjacent non-tumor tissues. Specifically, western blot analysis of eight paired CRC samples showed that ATG13 protein levels were higher in tumor tissues than in adjacent normal tissues (Fig. 8A). In addition, qPCR further showed a significant upregulation of ATG13 mRNA in CRC tissues, whereas no significant upregulation was observed in adjacent non-tumor tissues (Fig. 8B). Consistent with these results, the mRNA and protein expression levels of ATG13 in CRC cell lines were also significantly higher than those in normal intestinal epithelial cells (Fig. 8C, D). The protein level of ATG13 in 10 cases of colorectal cancer tissue and 10 cases of non-cancer tissue were detected using IHC experiment. IHC analysis revealed the expression level of ATG13 in tumor tissues is significantly higher than that in adjacent normal tissues (Fig. 8E).

To investigate the role of ATG13 in CRC, we silenced ATG13 in high-expression HCT116 cells using siRNA, confirming the knockdown with Western blotting and qPCR (Fig. S5C). To further explore the contribution of ATG13 to CRC progression, we co-cultured HCT116 cells with CAFs-conditioned media (CAFs CM). Growth curve and colony formation assays showed that si-ATG13 significantly reduced CRC cell proliferation (Figs. 8F, G, S5D). Next, Transwell assays demonstrated that si-ATG13 inhibited CRC cell migration and invasion (Figs. 8H, S5E). Consistently, Western blot analysis revealed that, upon ATG13 knockdown, Vimentin and N-Cadherin expression decreased significantly, while E-Cadherin increased (Fig. 8I). Finally, to evaluate the in vivo tumorigenic function of ATG13, we used a subcutaneous xenograft model, where tumors from HCT116-shATG13 mice exhibited reduced volume and weight compared to controls, consistent with our in vitro findings (Fig. 8J).

### ATG13 expressed in iCAFs acts as a negative regulator of ferroptosis in CRC cells

**Fig. 9:**
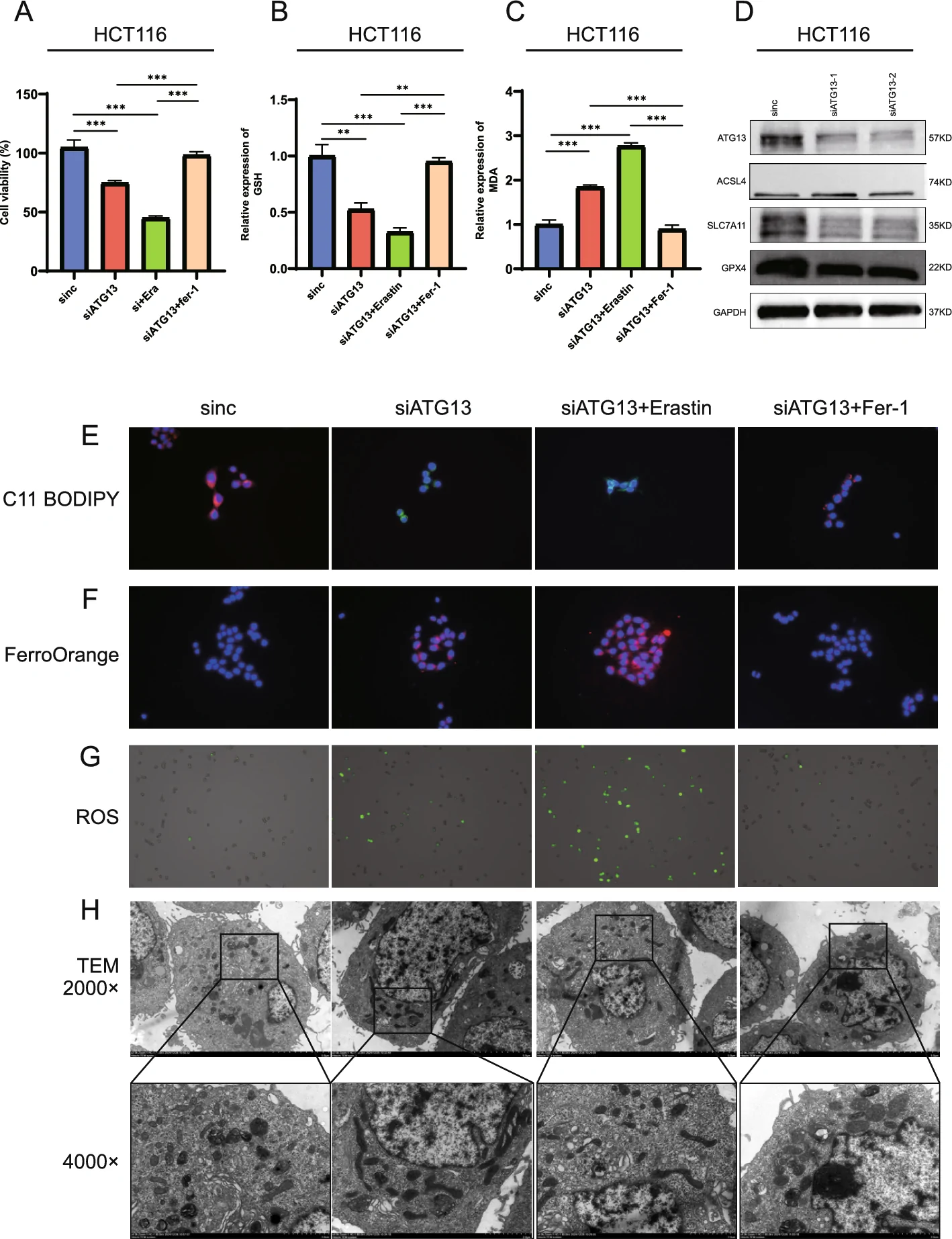
iCAFs-enriched ATG13 acts as a negative regulator of ferroptosis in CRC cells. **A** CCK-8 assay-based growth curve analysis of cells treated with Erastin. **B** Measurement of GSH levels in Erastin-treated cells. **C** Measurement of MDA levels in Erastin-treated cells. **D** Western blot analysis reveals changes in ferroptosis marker protein expression in CRC cells transfected with specific plasmids. **E** Western blot analysis reveals expression of lipid peroxidation markers in CRC cells transfected with specific plasmids. **F** Western blot analysis reveals expression of ferrous iron in CRC cells transfected with specific plasmids. **G** Detection of ROS levels in Erastin-treated cells. Scale bar: 100 µm. **H** Erastin treatment of specified cells followed by transmission electron microscopy (TEM) analysis. Scale bars: 500 nm (first row) and 100 nm (second row)

Ferroptosis is an iron-dependent regulated cell death induced by lipid peroxidation [4, 5]. To determine whether ATG13 modulates ferroptosis, we assessed the viability of HCT116 cells treated with the ferroptosis inducer erastin. As shown in Fig. 9A, si-ATG13 enhanced the growth inhibition of CRC cells induced by erastin, while the ferroptosis inhibitor ferrostatin-1 significantly reversed the growth suppression caused by si-ATG13. To further validate this observation, we treated si-ATG13 HCT116 cells with erastin and observed increased accumulation of MDA, BODIPY C11, Fe^2^⁺, and ROS, reduced accumulation of GSH, all of which were reversed by ferrostatin-1 (Fig. 9B–G). Transmission electron microscopy (TEM) analysis further revealed that si-ATG13-treated HCT116 cells exhibited typical ferroptotic morphological features, including mitochondrial shrinkage and increased membrane density, which were protected by ferrostatin-1 (Fig. 9H). Additionally, Western blot analysis of ferroptosis biomarkers showed that ATG13 knockdown downregulated GPX4 and SLC7A11 at both the protein and mRNA levels, whereas ACSL4 was upregulated (Fig. 9D). These results further support ATG13 as a negative regulator of ferroptosis.

## Discussion

To improve personalized treatment for CRC, a deeper understanding of the clinical subtypes and TME heterogeneity is essential. Therefore, in this study, we utilized single-cell sequencing combined with spatial transcriptomics to comprehensively investigate the cellular composition and spatial architecture of the CRC tumor microenvironment. Specifically, we focused on CAF subtypes and further explored the regulatory relationship between the core CAF subpopulation and ferroptosis, identifying CAF-related ferroptosis genes. By leveraging single-cell and spatial transcriptomics, we subsequently discovered that ATG13 enrichment in CAFs is significantly associated with the malignant progression of CRC.

CAFs are the most common stromal cells in solid tumors, forming a variety of highly plastic clusters that exhibit heterogeneity in response to changes in the TME [31, 32]. In recent years, the advent of scRNA-seq has enabled the identification of increasingly diverse CAFs subpopulations, including myCAFs, iCAFs, apCAFs, etc. [33]. In this study, we identified five CAF subtypes: apCAFs, iCAFs, mCAFs, vCAFs, and FSTL5+ Fibroblasts, the latter of which is a novel CAF subpopulation whose role in the CRC microenvironment remains unexplored and warrants further investigation. We observed that the proportion of malignant endothelial cells (EC) was relatively high in CRC tissues. Given the pivotal role of CAFs in regulating malignant epithelial cells, we further performed cellular network analysis, which revealed an increased proportion of iCAFs and mCAFs subpopulations. Notably, we found an interaction between the iCAFs and mCAFs subgroups. However, prior studies have not addressed the interactions between these two subpopulations. iCAFs mainly contribute to shaping an immunosuppressive microenvironment, and in breast cancer patients receiving PD-1 immunotherapy, iCAFs demonstrate an enhanced ability to promote cancer cell proliferation, drive EMT, and facilitate the formation of an immunosuppressive microenvironment [34]. In our study, we discovered that iCAFs are involved not only in regulating the non-canonical Wnt signaling pathway but also in regulating mesenchymal cell differentiation and metal ion transport. Ma et al. found that marker gene in situ hybridization showed that mCAFs and iCAFs are distinct CAF populations. mCAFs are present in all tumors but play a significant role at the tumor-stroma boundary [35]. Our study also showed that mCAFs act as signaling senders, secreting specific ligands to bind receptors on iCAFs, thereby driving tumor progression. KEGG enrichment analysis revealed that mCAFs were significantly enriched in biological processes, including cell–matrix adhesion. mCAFs synthesize extracellular matrix (ECM) and may restrict T-cell invasion in low-grade tumors by encapsulating tumor nests. iCAFs, on the other hand, accumulate in late-stage, highly invasive tumors and express high levels of cytokines and chemokines to facilitate the recruitment and activation of immune cells. These findings highlight the key roles of mCAFs and iCAFs in the CAFs population, where mCAFs drive tumor survival and progression through high ECM activity, while iCAFs contribute to the immunosuppressive microenvironment, together facilitating cancer progression.

Gastrointestinal tumors are highly malignant, with a complex molecular composition and strong TME heterogeneity, lacking universal therapeutic targets, and commonly exhibit immune evasion. Thus, it is critical to explore new therapeutic targets for gastrointestinal cancers and identify novel strategies to overcome current immune therapy resistance. Tumor development is not solely dependent on epigenetic modifications in cancer cells but is also influenced by the components of the TME. During the anti-tumor immune response, complex crosstalk occurs between ferroptotic cancer cells and immune cells. In breast cancer immunotherapy, blocking the pS337 mutation of PSAT1 using CPP enhances IFNγ-induced ferroptosis and improves the efficacy of PD-1 therapy in TNBC [36]. Stromal cells in the tumor microenvironment typically promote tumor progression and drug resistance. CAFs can modulate ferroptosis in TME tumor cells. CAFs secrete cysteine via the TGF-β/SMAD3/ATF4 signaling axis, thereby enhancing the extracellular cysteine supply to support GSH synthesis and induce resistance to ferroptosis in pancreatic ductal adenocarcinoma cells [37]. Conversely, ferroptosis in tumor cells can also affect CAFs' function. Overexpression of Anoctamin 1 (ANO1) correlates with increased CAF abundance and poor immune therapy outcomes. ANO1 inhibits tumor cell ferroptosis by regulating the PI3K-Akt signaling axis, promoting the production and secretion of TGF-β by cancer cells, recruiting CAFs, and conferring resistance to immunotherapy [38].

Our study reveals that only one CAFs-related gene, ATG13, is significantly differentially expressed in CRC patients and inversely correlates with overall survival (OS). Previous studies have shown that ATG13, a core member of the autophagy initiation complex, is not only involved in autophagy regulation but also closely associated with ferroptosis [39]. Our in vitro and in vivo experiments demonstrated that si-ATG13 inhibits CRC cell proliferation, migration, and invasion. Using single-cell sequencing combined with spatial transcriptomics, we identified high expression of ferroptosis-related genes in both epithelial and fibroblast subpopulations. After reclustering fibroblasts, we identified five CAF subpopulations. GSVA analysis revealed that the iCAFs subgroup exhibited higher enrichment in lipid metabolism, iron ion transport, and lipid oxidation pathways associated with ferroptosis. To further define the interaction patterns among fibroblast populations, CellChat analysis indicated strong interactions between mCAFs and iCAFs. To explore the deeper relationship between iCAFs and ferroptosis, we applied WGCNA and found that the Hub genes in the iCAFs-M10 co-expression module showed the strongest correlation with ferroptosis-related genes, with only ATG13 showing an inverse correlation with poor prognosis. These results support the involvement of ATG13 in the iCAFs-mCAFs interaction network, possibly modulating tumor microenvironment remodeling via ferroptosis-related signaling pathways. Using spatial transcriptomics, we discovered that ATG13 is highly expressed in the fibroblast region, and mIHC results confirmed its significant overexpression in CAF-enriched areas. By detecting GPX4, SLC7A11, ACSL4, MDA, Fe^2^⁺, GSH, and lipid peroxidation levels, we found that si-ATG13 induced ferroptosis. More importantly, these changes induced by si-ATG13 were inhibited by the ferroptosis inhibitor Ferrostatin-1. Our findings suggest that ATG13 functions as a negative regulator of ferroptosis in CRC tumor cells, and its spatial enrichment in iCAF regions raises the possibility that ATG13, spatially associated with CAF regions may contribute to tumor progression. However, the precise paracrine or juxtacrine mechanisms by which iCAF-expressed ATG13 influences tumor cell ferroptosis remain to be directly demonstrated.

In summary, this study integrates scRNA-seq and spatial transcriptomics technologies to comprehensively elucidate the heterogeneity and spatial distribution of CAFs within the tumor microenvironment. We further investigate interactions between CAF subpopulations and identify ferroptosis-related CAF genes using WGCNA. However, this study has several limitations. First, the scRNA-seq and ST data used in this study were obtained from public datasets, although they encompass a large volume of single-cell data; more internal scRNA-seq and ST datasets are needed. Although we have validated the oncogenic function of ATG13 in inhibiting ferroptosis in vitro and in vivo, the pro-tumor molecular mechanism of ATG13 that is spatially associated with CAF regions in colorectal cancer (CRC) remains to be investigated. Therefore, subsequent studies will focus on elucidating the potential mechanism by which ATG13 inhibits ferroptosis.

## Conclusion

In summary, our study demonstrates that ATG13 is a potential diagnostic and prognostic biomarker associated with ferroptosis and CAFs. Knockdown of ATG13 induces ferroptosis in CRC cells and suppresses proliferation, migration, and invasion, supporting ATG13 as a potential therapeutic target. The spatial co-enrichment of ATG13 with iCAF markers suggests a stromal contribution to ferroptosis resistance in CRC. We acknowledge that while our transcriptomic and spatial data strongly implicate ATG13, spatially associated with CAF regions in ferroptosis regulation within the CRC TME, direct experimental evidence for the paracrine transfer or secretory mechanism of ATG13 from CAFs to tumor cells is currently lacking. Future studies employing CAF-specific ATG13 manipulation and co-culture systems will be essential to establish this mechanistic link.

## Supplementary Information

Additional file 1. Figure S1. Patient-level pseudo-bulk analysis.

Additional file 2. Figure S2. Expression of the 4 genes and survival analysis.

Additional file 3. Figure S3. Transcription factor regulatory network analysis and Molecular docking.

Additional file 4. Figure S4. Pearson correlation analysis to smooth CAFs score and ATG13 expression.

Additional file 5. Figure S5. Quantitative analysis. **A** Quantitative analysis of immunofluorescence co-localization of ATG13 and Vimentin. **B** Quantitative analysis of immunofluorescence co-localization of ATG13 and α-SMA. **C** qRT-PCR and WB analysis of siATG13. **D** Colony count analysis. **E** Transwell migration, Matrigel invasion assays, and EDU assay analysis.

Additional file 6.

## Data Availability

The datasets analyzed during the current study have been deposited in the [Gene Expression Omnibus] and are publicly accessible under the accession numbers [GSE166555, GSE146771, GSE132465, GSE144735, GSE188711]. The data can be accessed at the following permanent link: [https://www.ncbi.nlm.nih.gov/geo/]. The datasets analyzed during the current study have been deposited in the [Functional Genomics] and are publicly accessible under the accession number [EMTAB8107]. The data can be accessed at the following permanent link: [https://www.ebi.ac.uk/]. All accession numbers and associated files have been fully released and are available for verification.
