# Supplementary material for: Integrative single-cell and spatial transcriptomic analysis reveals that ATG13 enriched in cancer-associated fibroblasts is associated with the suppression of ferroptosis in colorectal cancer
Source: Discov Oncol. 2026 Jun 16;17:1234. doi: 10.1007/s12672-026-05386-2 (PMC13503995; doi:10.1007/s12672-026-05386-2)
Supplement: Supplementary file 1 [file 12672_2026_5386_MOESM1_ESM.pdf]

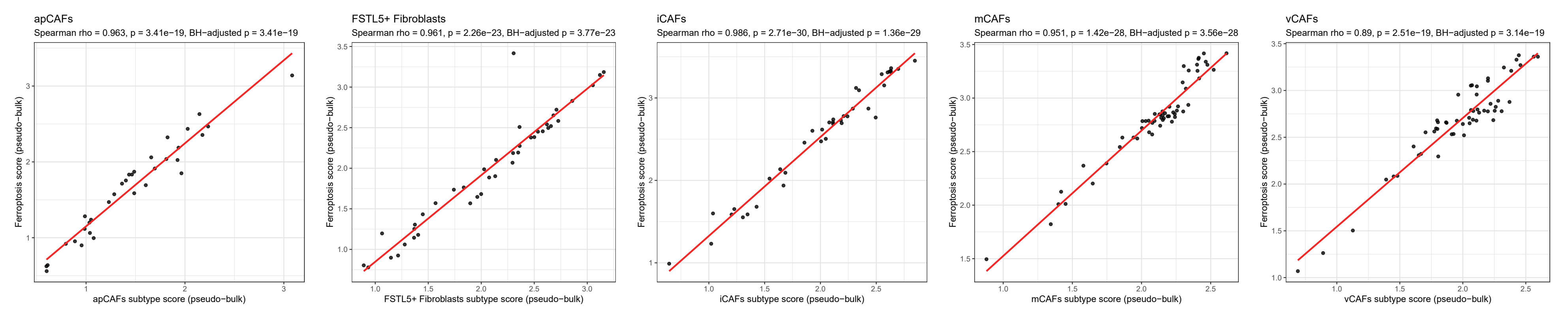
apCAFs
Spearman rho = 0.963, p = 3.41e−19, BH−adjusted p = 3.41e−19
Ferroptosis score (pseudo−bulk)
apCAFs subtype score (pseudo−bulk)
FSTL5+ Fibroblasts
Spearman rho = 0.961, p = 2.26e−23, BH−adjusted p = 3.77e−23
Ferroptosis score (pseudo−bulk)
FSTL5+ Fibroblasts subtype score (pseudo−bulk)
iCAFs
Spearman rho = 0.986, p = 2.71e−30, BH−adjusted p = 1.36e−29
Ferroptosis score (pseudo−bulk)
iCAFs subtype score (pseudo−bulk)
mCAFs
Spearman rho = 0.951, p = 1.42e−28, BH−adjusted p = 3.56e−28
Ferroptosis score (pseudo−bulk)
mCAFs subtype score (pseudo−bulk)
vCAFs
Spearman rho = 0.89, p = 2.51e−19, BH−adjusted p = 3.14e−19
Ferroptosis score (pseudo−bulk)
vCAFs subtype score (pseudo−bulk)
