# Supplementary material for: Integrative single-cell and spatial transcriptomic analysis reveals that ATG13 enriched in cancer-associated fibroblasts is associated with the suppression of ferroptosis in colorectal cancer
Source: Discov Oncol. 2026 Jun 16;17:1234. doi: 10.1007/s12672-026-05386-2 (PMC13503995; doi:10.1007/s12672-026-05386-2)
Supplement: Supplementary file 2 [file 12672_2026_5386_MOESM2_ESM.pdf]

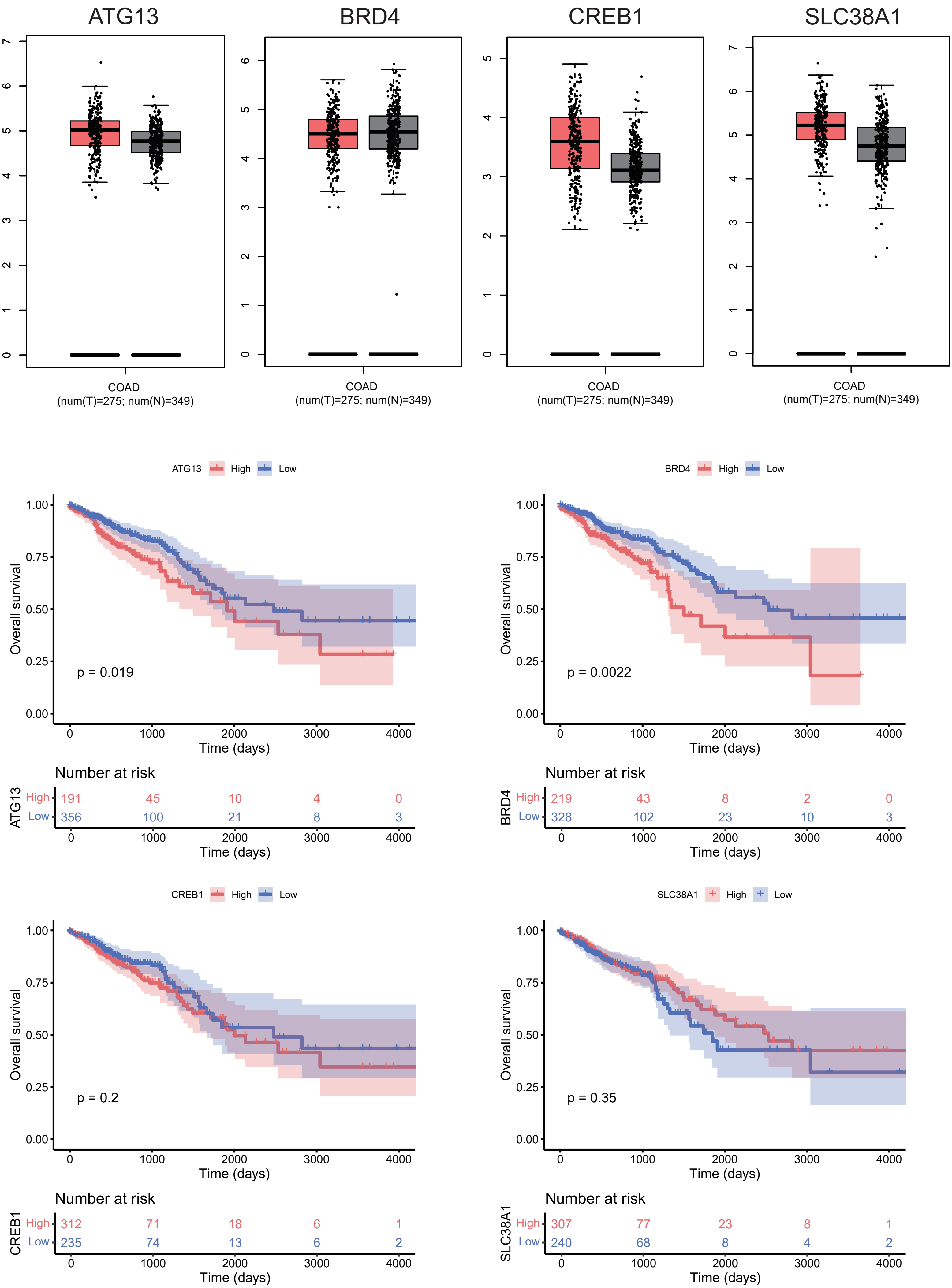
ATG13
BRD4
CREB1
SLC38A1
COAD
(num(T)=275; num(N)=349)
COAD
(num(T)=275; num(N)=349)
COAD
(num(T)=275; num(N)=349)
COAD
(num(T)=275; num(N)=349)
ATG13 High Low
Overall survival
p = 0.019
Time (days)
Number at risk
ATG13
High 191 45 10 4 0
Low 356 100 21 8 3
Time (days)
BRD4 High Low
Overall survival
p = 0.0022
Time (days)
Number at risk
BRD4
High 219 43 8 2 0
Low 328 102 23 10 3
Time (days)
CREB1 High Low
Overall survival
p = 0.2
Time (days)
Number at risk
CREB1
High 312 71 18 6 1
Low 235 74 13 6 2
Time (days)
SLC38A1 High Low
Overall survival
p = 0.35
Time (days)
Number at risk
SLC38A1
High 307 77 23 8 1
Low 240 68 8 4 2
Time (days)
