# Supplementary material for: Integrative single-cell and spatial transcriptomic analysis reveals that ATG13 enriched in cancer-associated fibroblasts is associated with the suppression of ferroptosis in colorectal cancer
Source: Discov Oncol. 2026 Jun 16;17:1234. doi: 10.1007/s12672-026-05386-2 (PMC13503995; doi:10.1007/s12672-026-05386-2)
Supplement: Supplementary file 3 [file 12672_2026_5386_MOESM3_ESM.pdf]

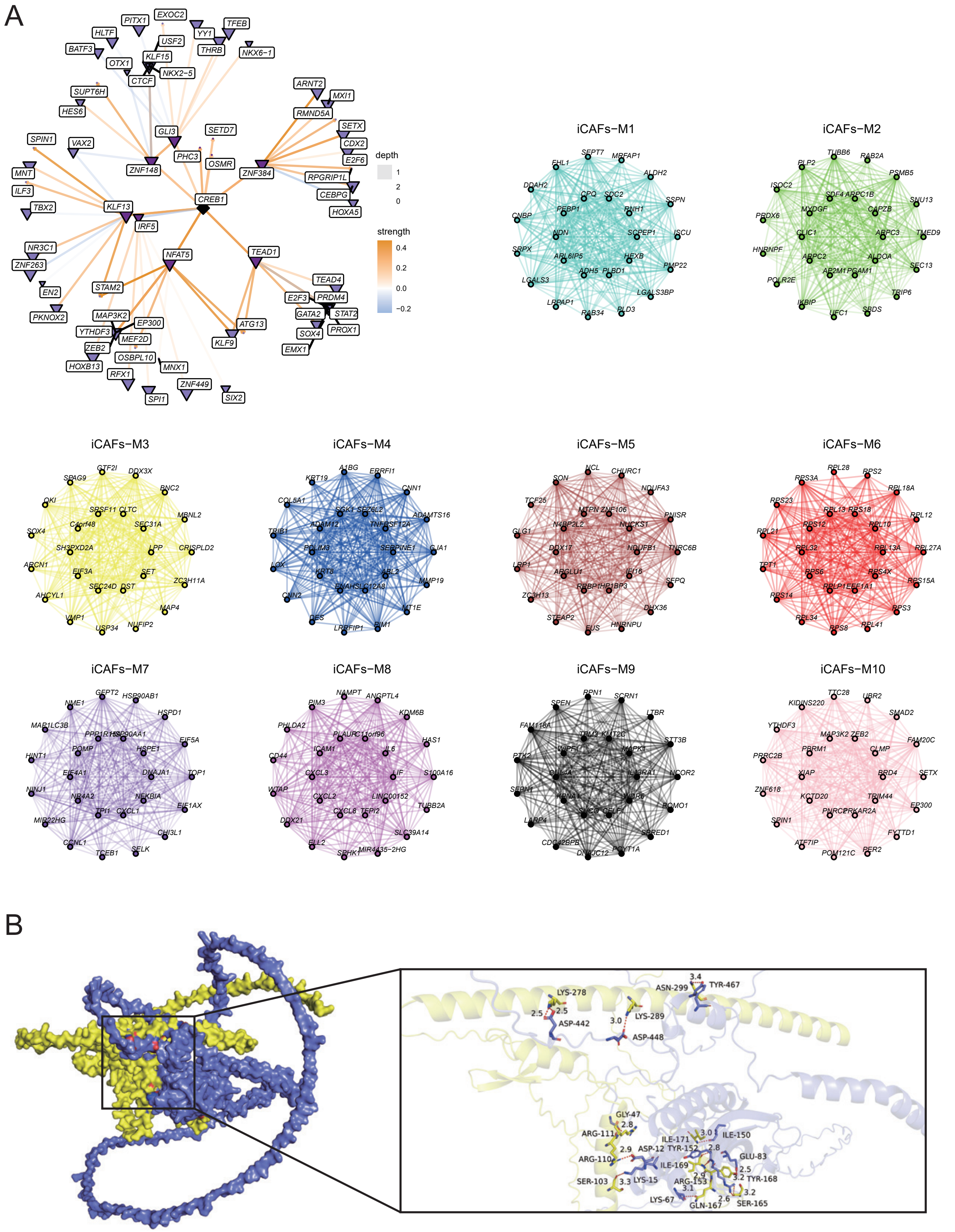
A
depth
1
2
0
strength
0.4
0.2
0.0
−0.2
CREB1
KLF13
NFAT5
TEAD1
ZNF148
ZNF384
iCAFs−M1
iCAFs−M2
iCAFs−M3
iCAFs−M4
iCAFs−M5
iCAFs−M6
iCAFs−M7
iCAFs−M8
iCAFs−M9
iCAFs−M10
B
LYS-278
ASP-442
ASN-299
TYR-467
LYS-289
ASP-448
GLY-47
ARG-111
ARG-110
SER-103
LYS-15
ILE-171
ASP-12
TYR-152
ILE-150
ILE-169
GLU-83
ARG-153
TYR-168
LYS-67
GLN-167
SER-165
