# Supplementary material for: Integrative single-cell and spatial transcriptomic analysis reveals that ATG13 enriched in cancer-associated fibroblasts is associated with the suppression of ferroptosis in colorectal cancer
Source: Discov Oncol. 2026 Jun 16;17:1234. doi: 10.1007/s12672-026-05386-2 (PMC13503995; doi:10.1007/s12672-026-05386-2)
Supplement: Supplementary file 4 [file 12672_2026_5386_MOESM4_ESM.pdf]

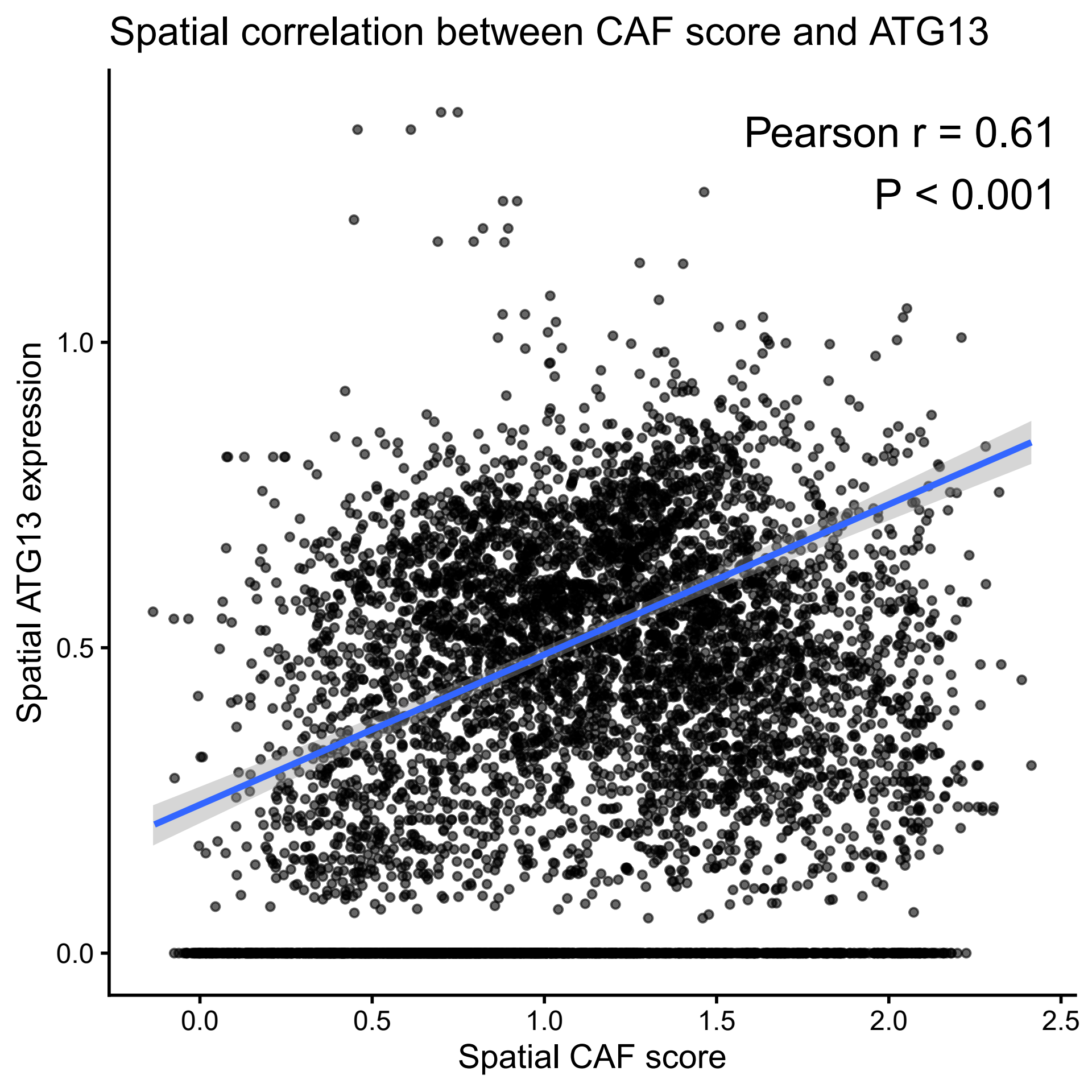
Spatial correlation between CAF score and ATG13
Pearson r = 0.61
P < 0.001
Spatial ATG13 expression
1.0
0.5
0.0
0.0
0.5
1.0
1.5
2.0
2.5
Spatial CAF score
