# Supplementary material for: Integrative single-cell and spatial transcriptomic analysis reveals that ATG13 enriched in cancer-associated fibroblasts is associated with the suppression of ferroptosis in colorectal cancer
Source: Discov Oncol. 2026 Jun 16;17:1234. doi: 10.1007/s12672-026-05386-2 (PMC13503995; doi:10.1007/s12672-026-05386-2)
Supplement: Supplementary file 5 [file 12672_2026_5386_MOESM5_ESM.pdf]

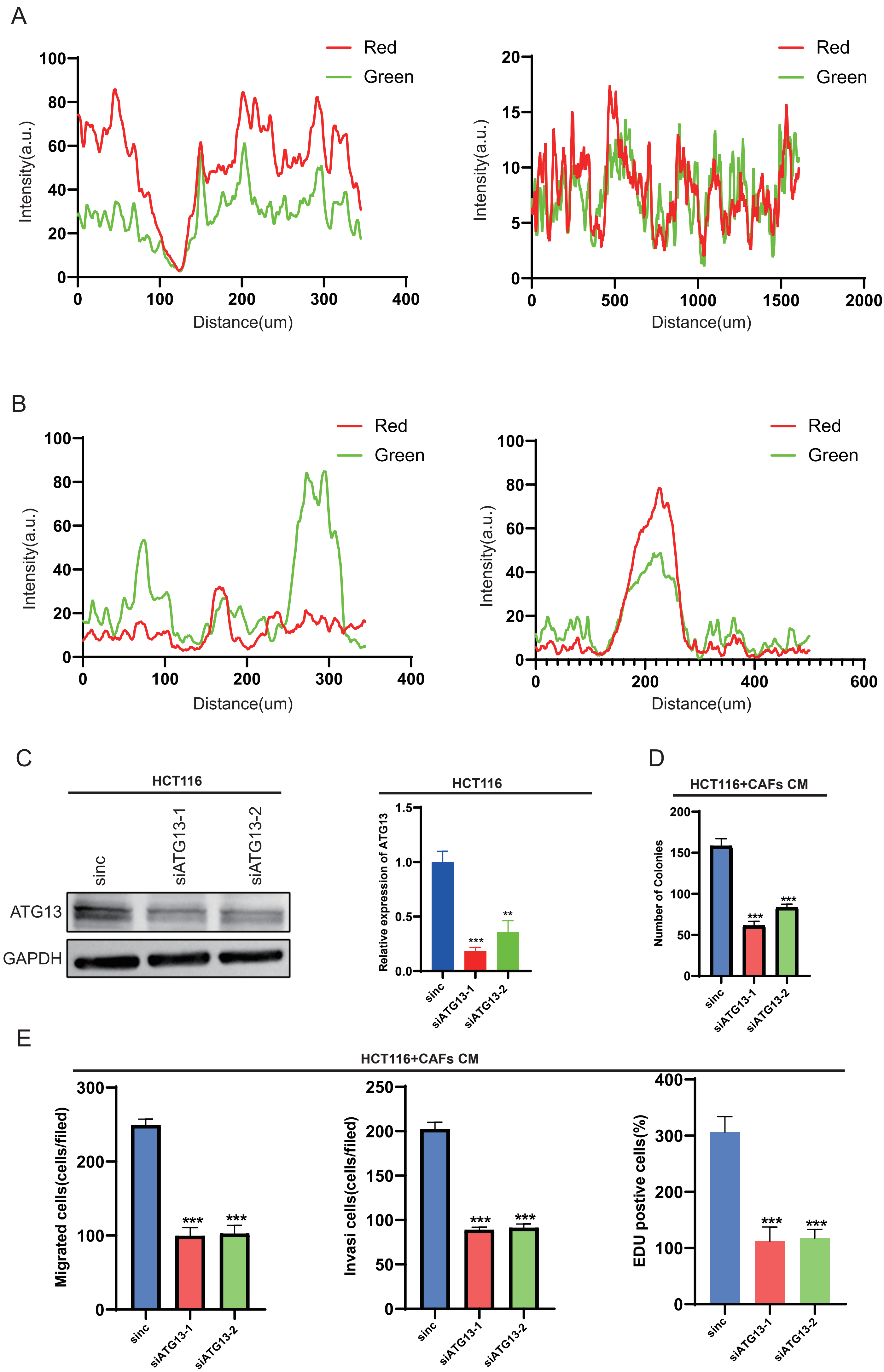

A
Red
Green
Intensity(a.u.)
Distance(um)
B
Red
Green
Intensity(a.u.)
Distance(um)
C
HCT116
sinc
siATG13-1
siATG13-2
ATG13
GAPDH
HCT116
Relative expression of ATG13
D
HCT116+CAFs CM
Number of Colonies
E
HCT116+CAFs CM
Migrated cells(cells/filed)
Invasi cells(cells/filed)
EDU postive cells(%)
