# Supplementary material for: Integrative single-cell and spatial transcriptomic analysis reveals that ATG13 enriched in cancer-associated fibroblasts is associated with the suppression of ferroptosis in colorectal cancer
Source: Discov Oncol. 2026 Jun 16;17:1234. doi: 10.1007/s12672-026-05386-2 (PMC13503995; doi:10.1007/s12672-026-05386-2)
Supplement: Supplementary file 6 [file 12672_2026_5386_MOESM6_ESM.pdf]

Figure8A-1

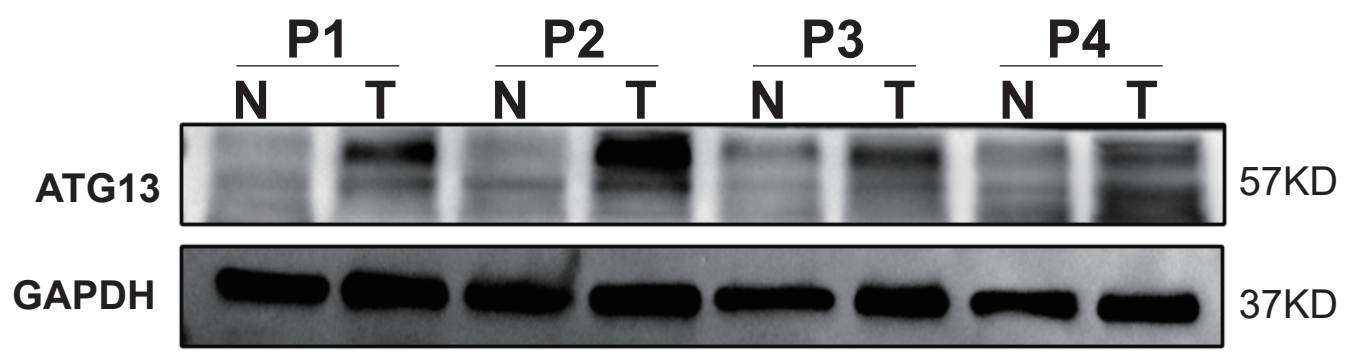

P1
P2
P3
P4
N T N T N T N T
ATG13
GAPDH
57KD
37KD

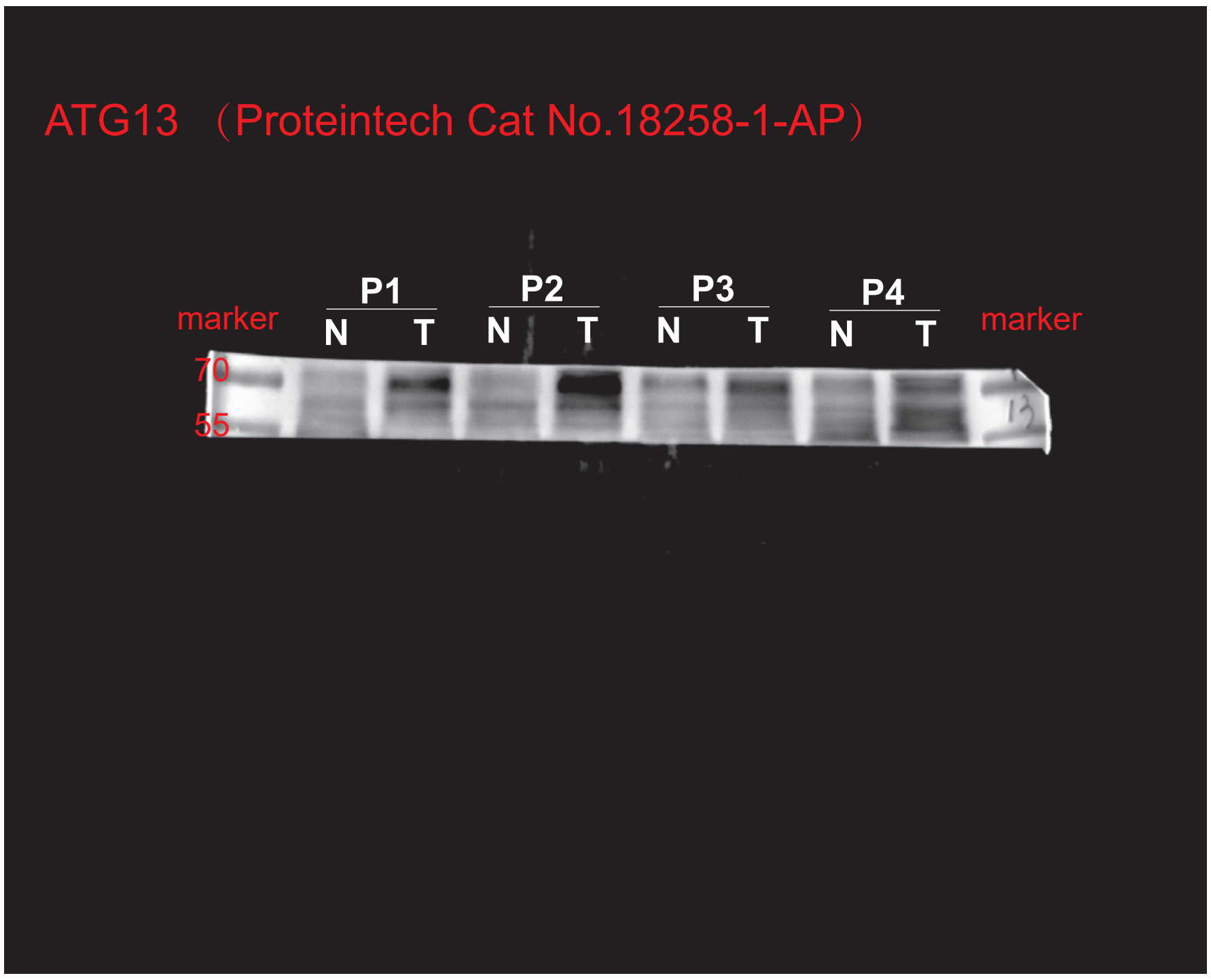

ATG13 （Proteintech Cat No.18258-1-AP）
marker
P1
P2
P3
P4
N T N T N T N T
marker
70
55

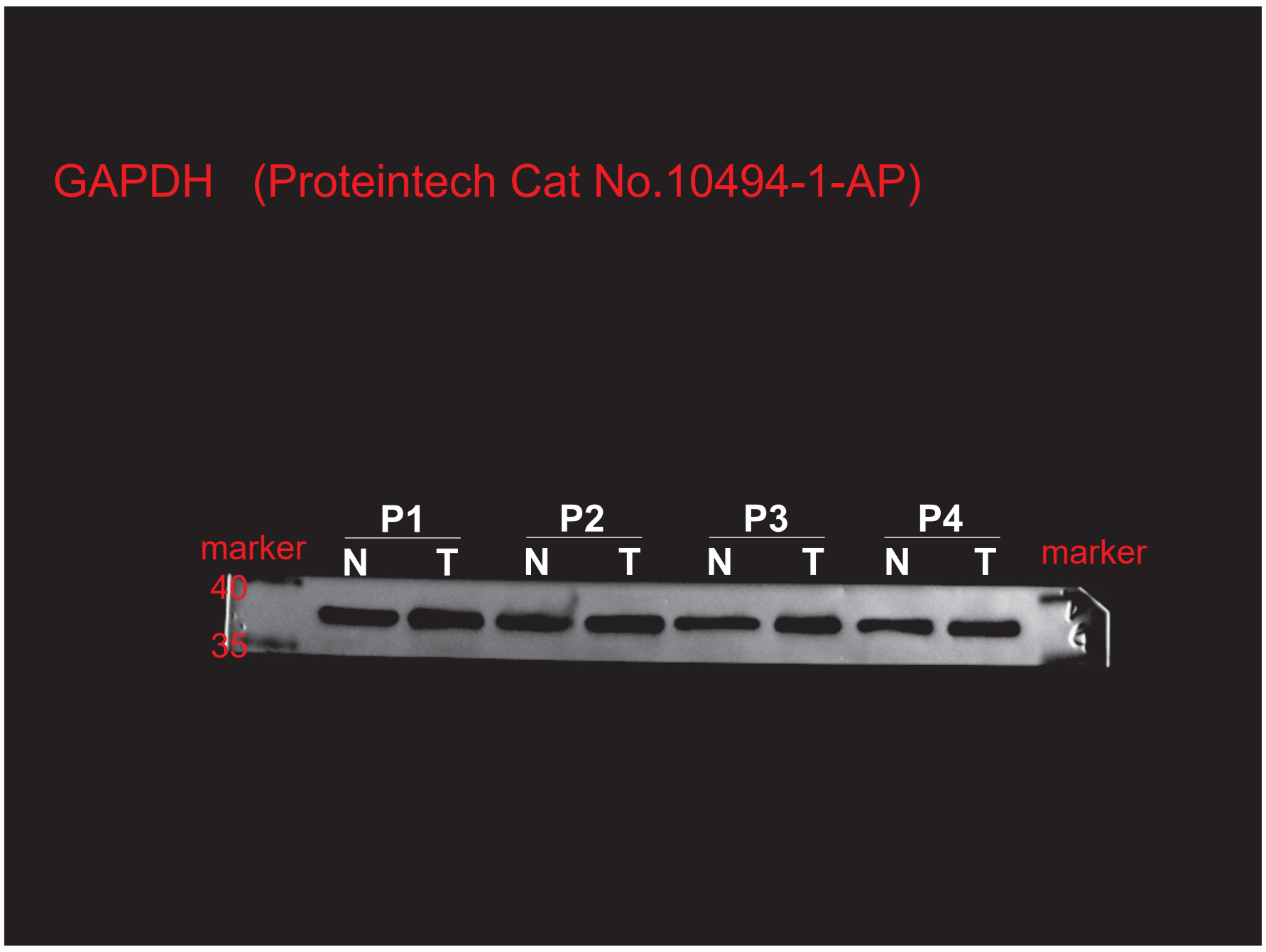

GAPDH (Proteintech Cat No.10494-1-AP)
marker
P1
P2
P3
P4
N T N T N T N T
marker
40
35

Figure8A-2

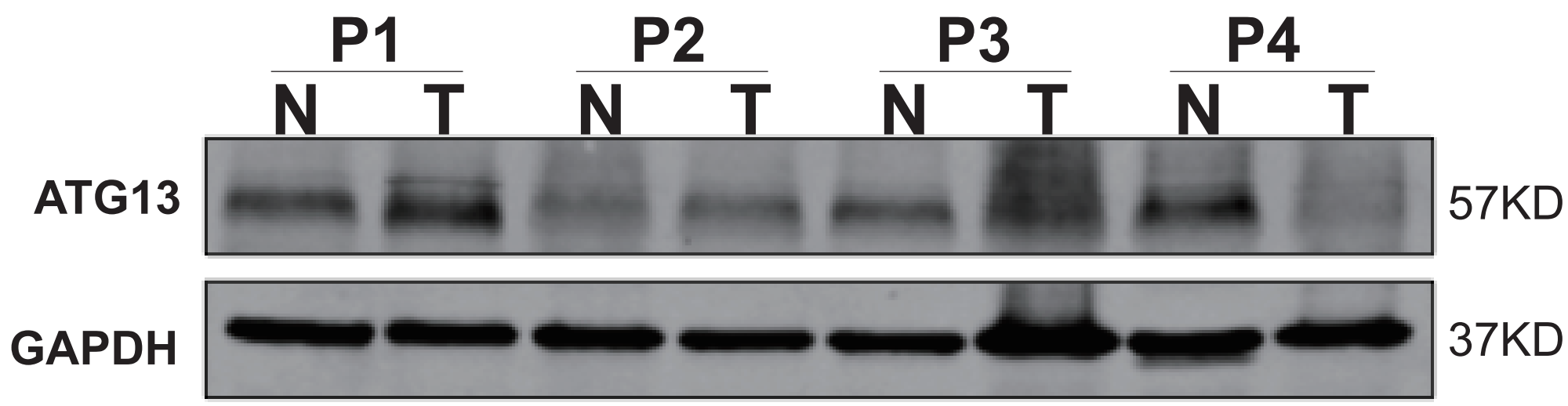
P1
P2
P3
P4
N
T
N
T
N
T
N
T
ATG13
57KD
GAPDH
37KD

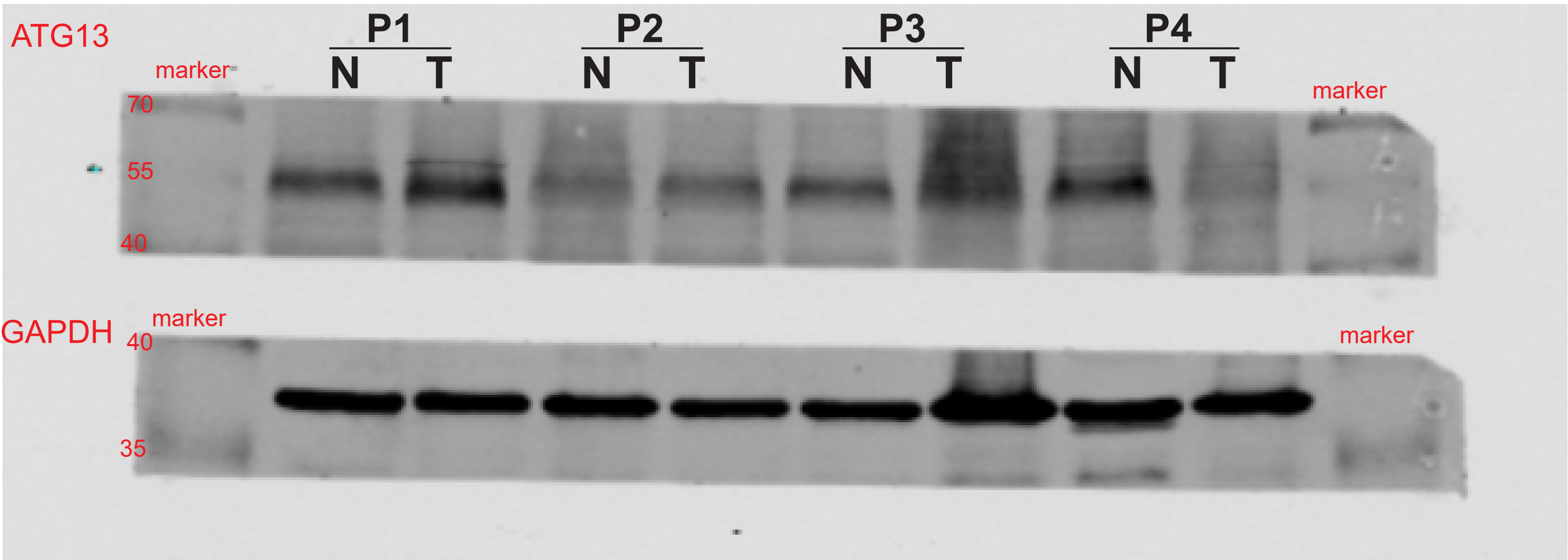
ATG13
marker
P1
P2
P3
P4
N
T
N
T
N
T
N
T
marker
70
55
40
GAPDH
marker
40
35
marker

Figure8D

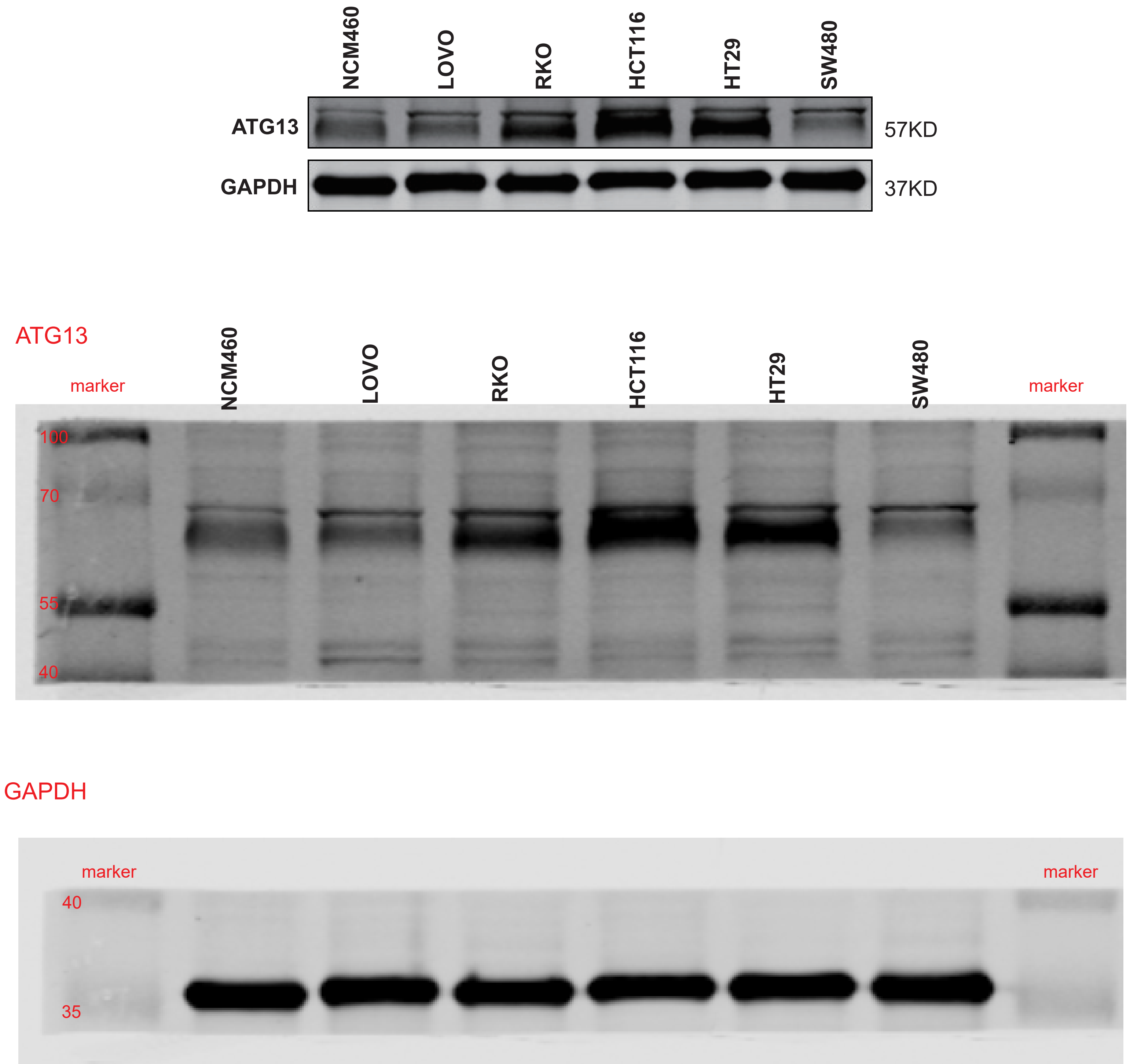
NCM460
LOVO
RKO
HCT116
HT29
SW480
ATG13
57KD
GAPDH
37KD
ATG13
marker
NCM460
LOVO
RKO
HCT116
HT29
SW480
marker
100
70
55
40
GAPDH
marker
marker
40
35

Figure8I

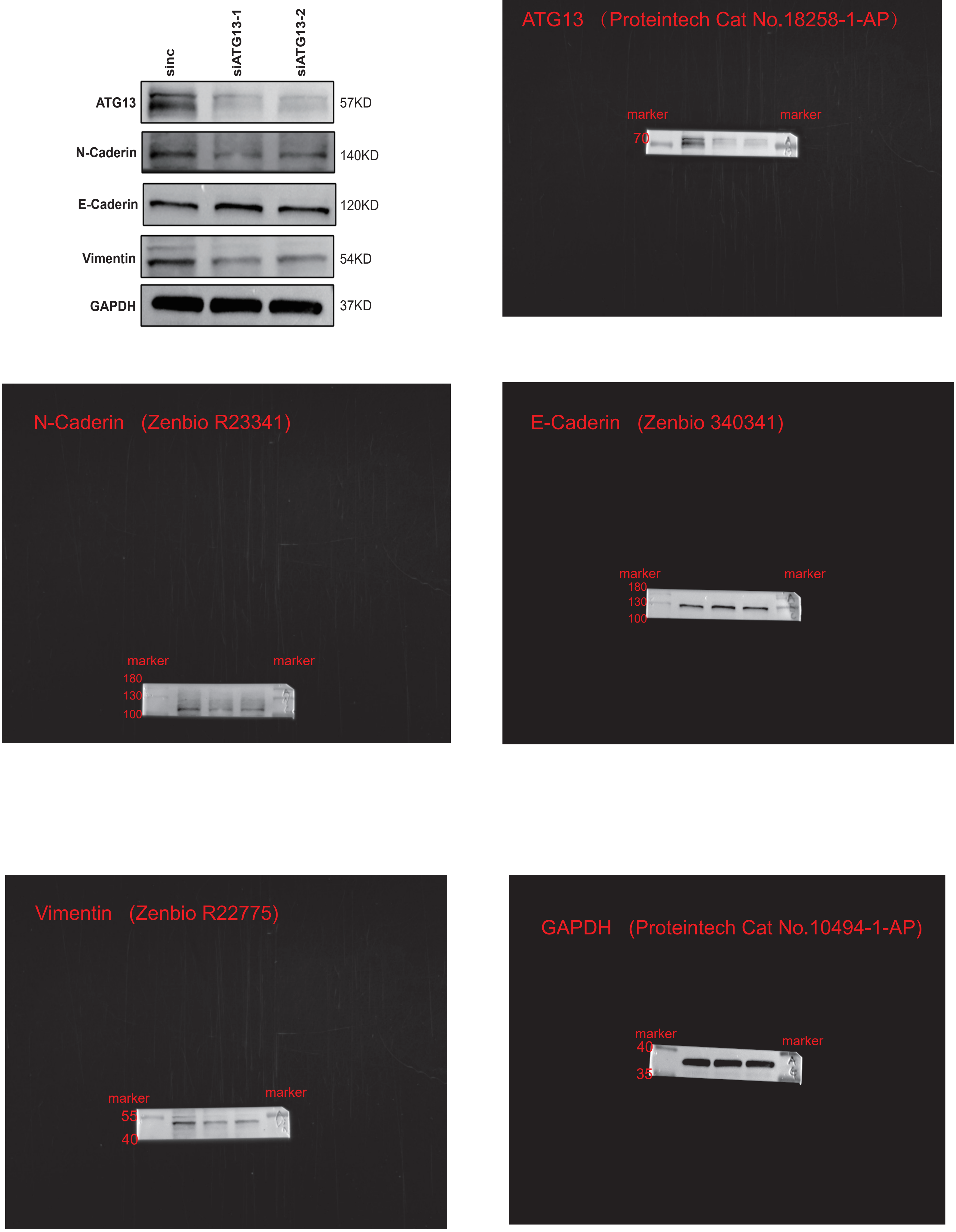

sinc
siATG13-1
siATG13-2
ATG13
57KD
N-Caderin
140KD
E-Caderin
120KD
Vimentin
54KD
GAPDH
37KD
ATG13 （Proteintech Cat No.18258-1-AP）
marker
marker
70
N-Caderin (Zenbio R23341)
marker
marker
180
130
100
E-Caderin (Zenbio 340341)
marker
marker
180
130
100
Vimentin (Zenbio R22775)
marker
marker
55
40
GAPDH (Proteintech Cat No.10494-1-AP)
marker
marker
40
35

Figure9D

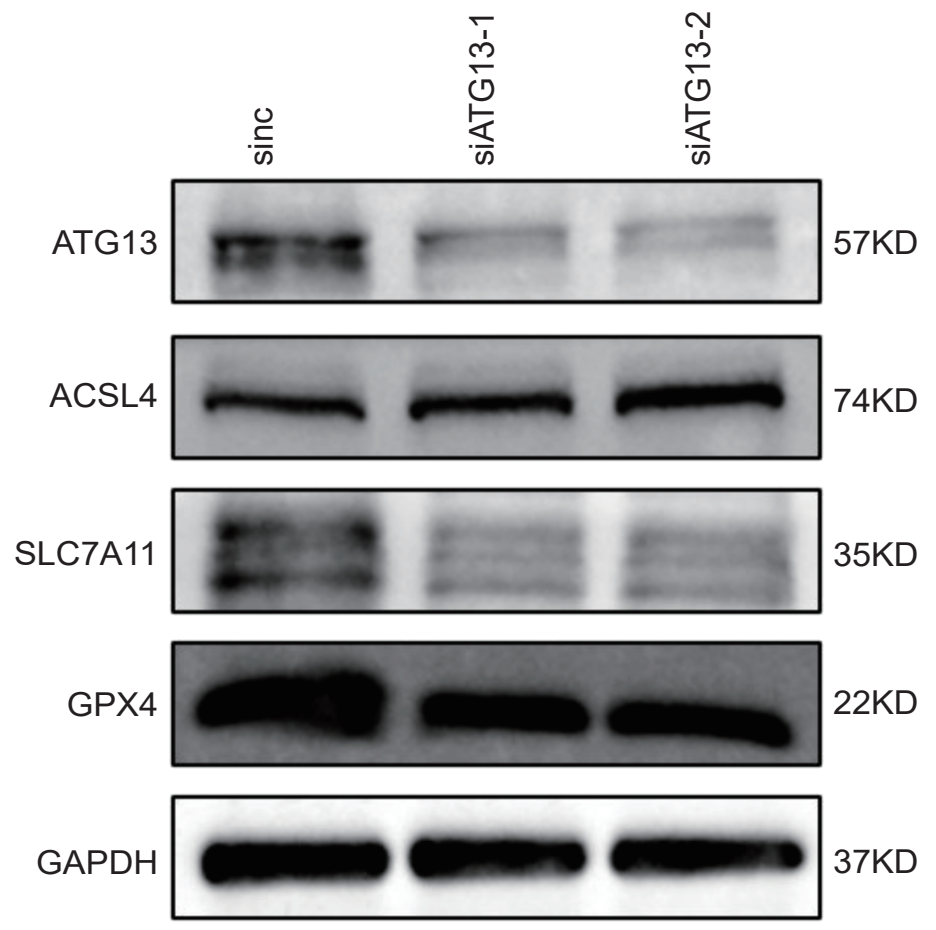
sinc
siATG13-1
siATG13-2
ATG13
57KD
ACSL4
74KD
SLC7A11
35KD
GPX4
22KD
GAPDH
37KD

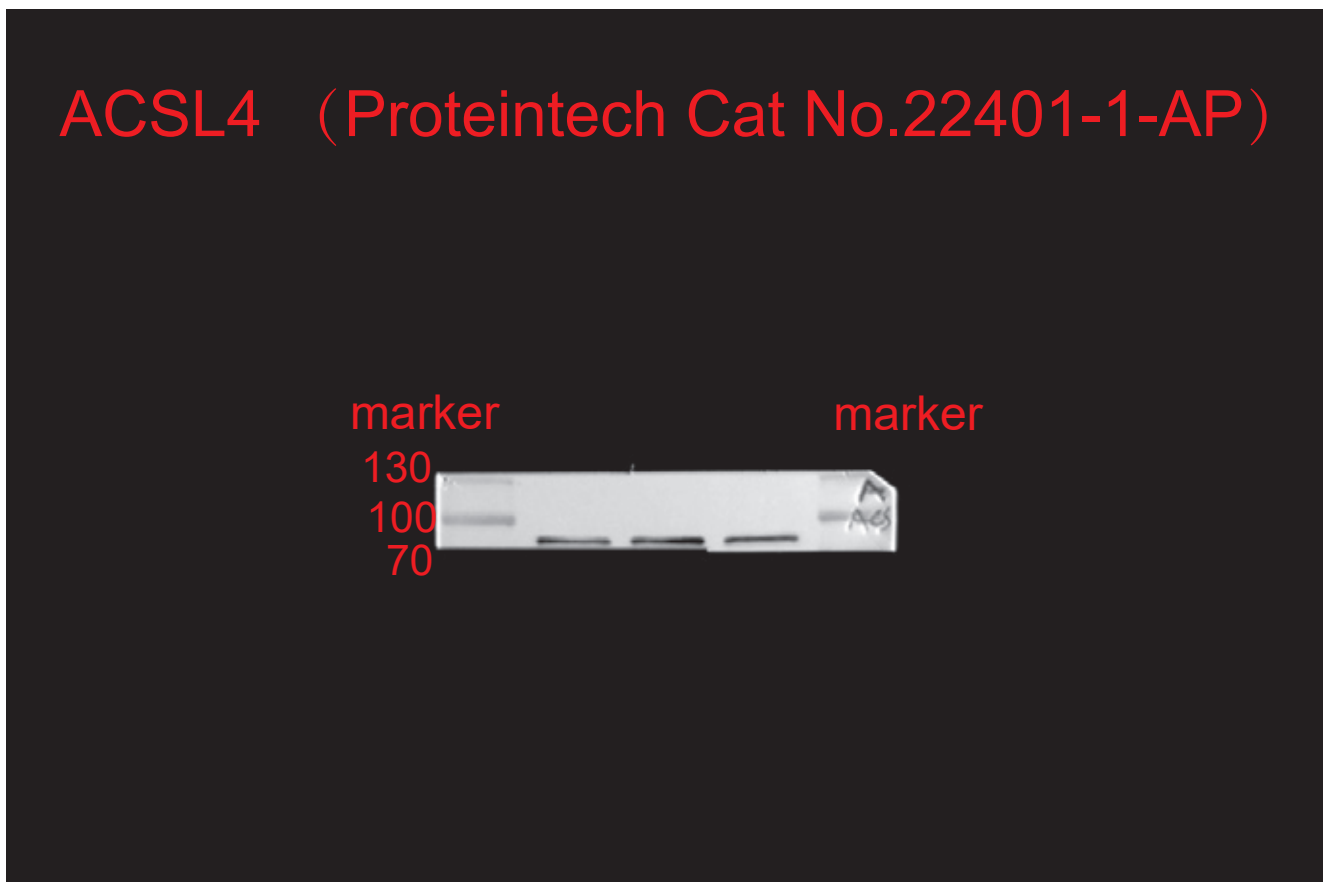
ACSL4 （Proteintech Cat No.22401-1-AP）
marker
marker
130
100
70

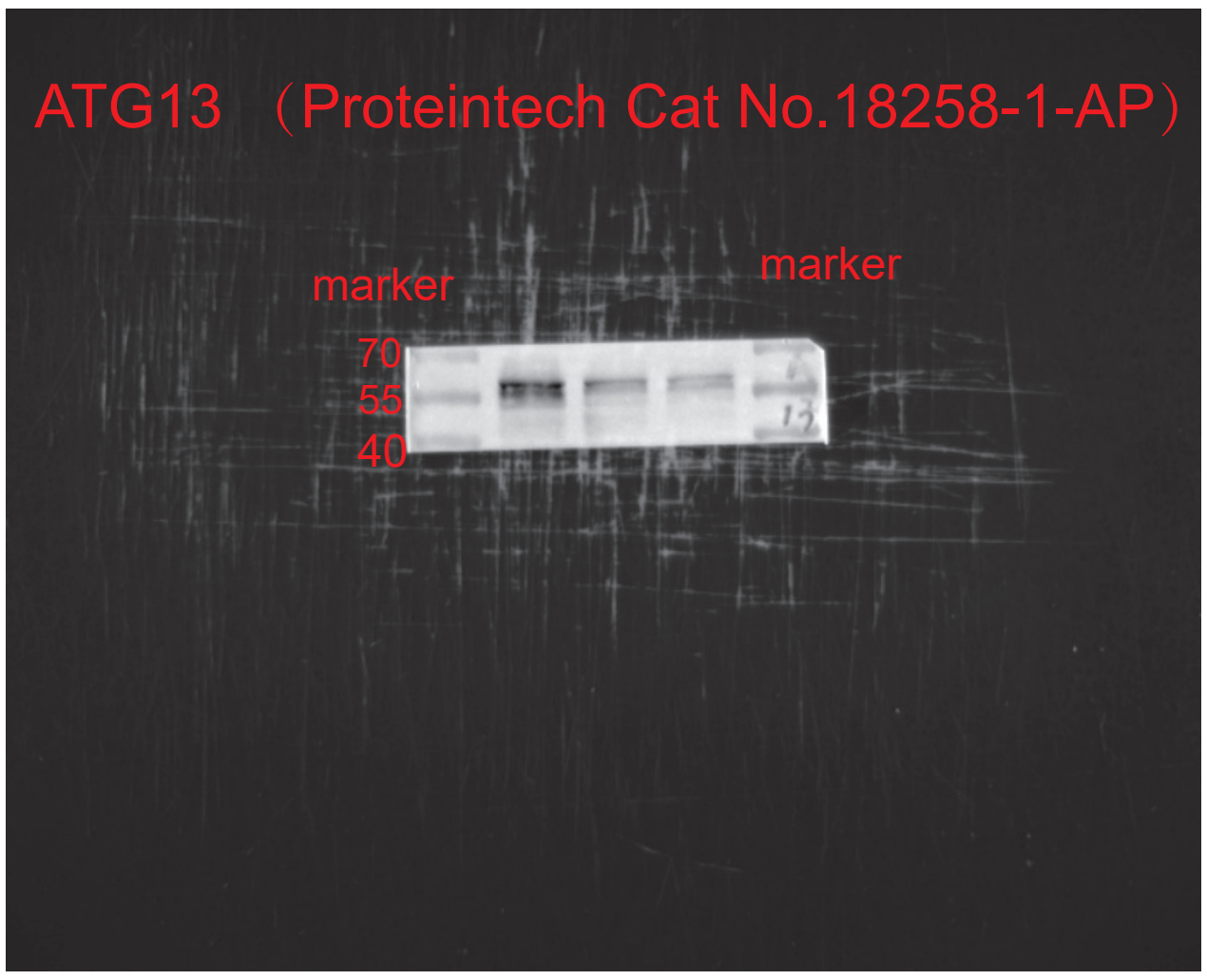
ATG13 （Proteintech Cat No.18258-1-AP）
marker
marker
70
55
40

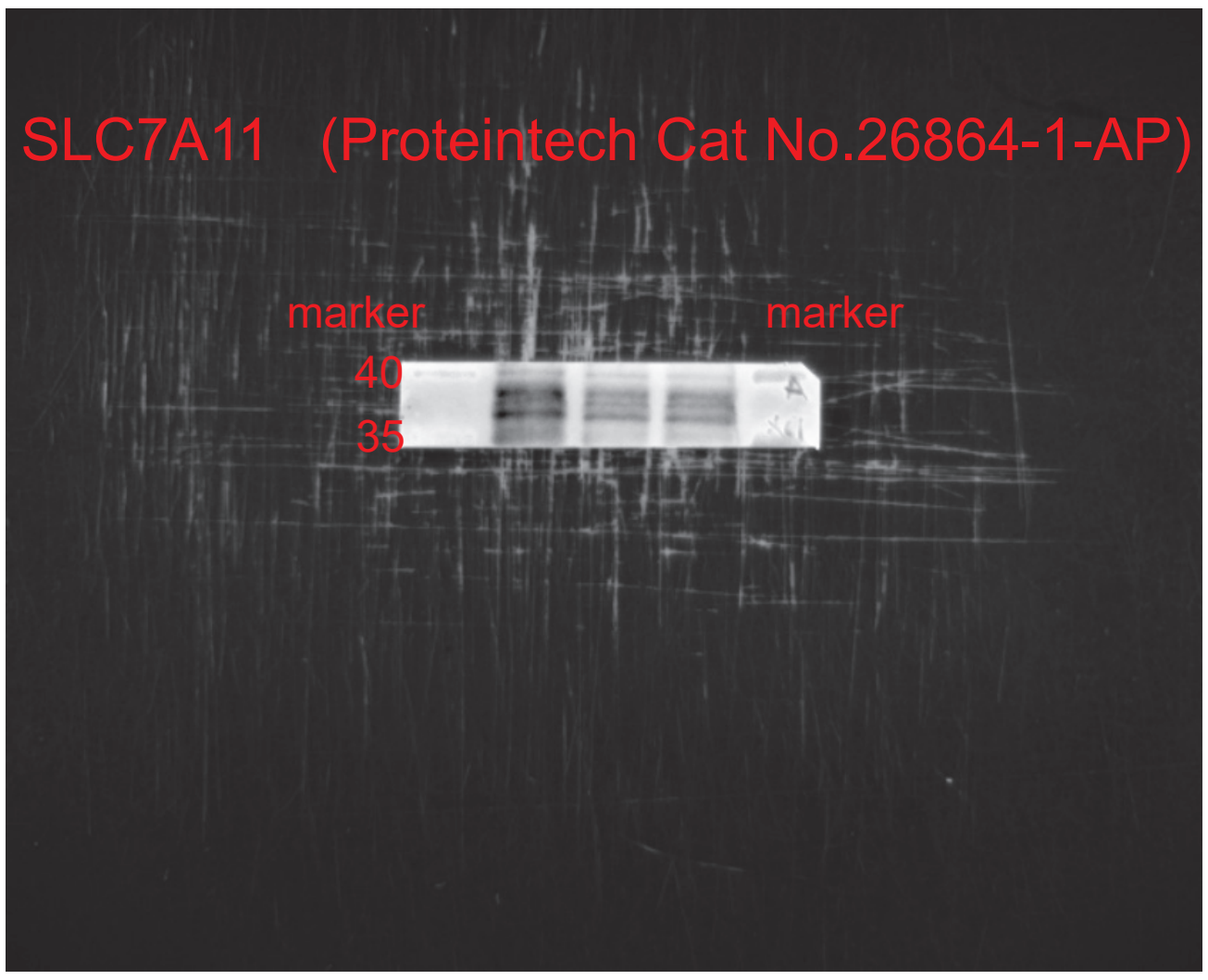
SLC7A11 (Proteintech Cat No.26864-1-AP)
marker
marker
40
35

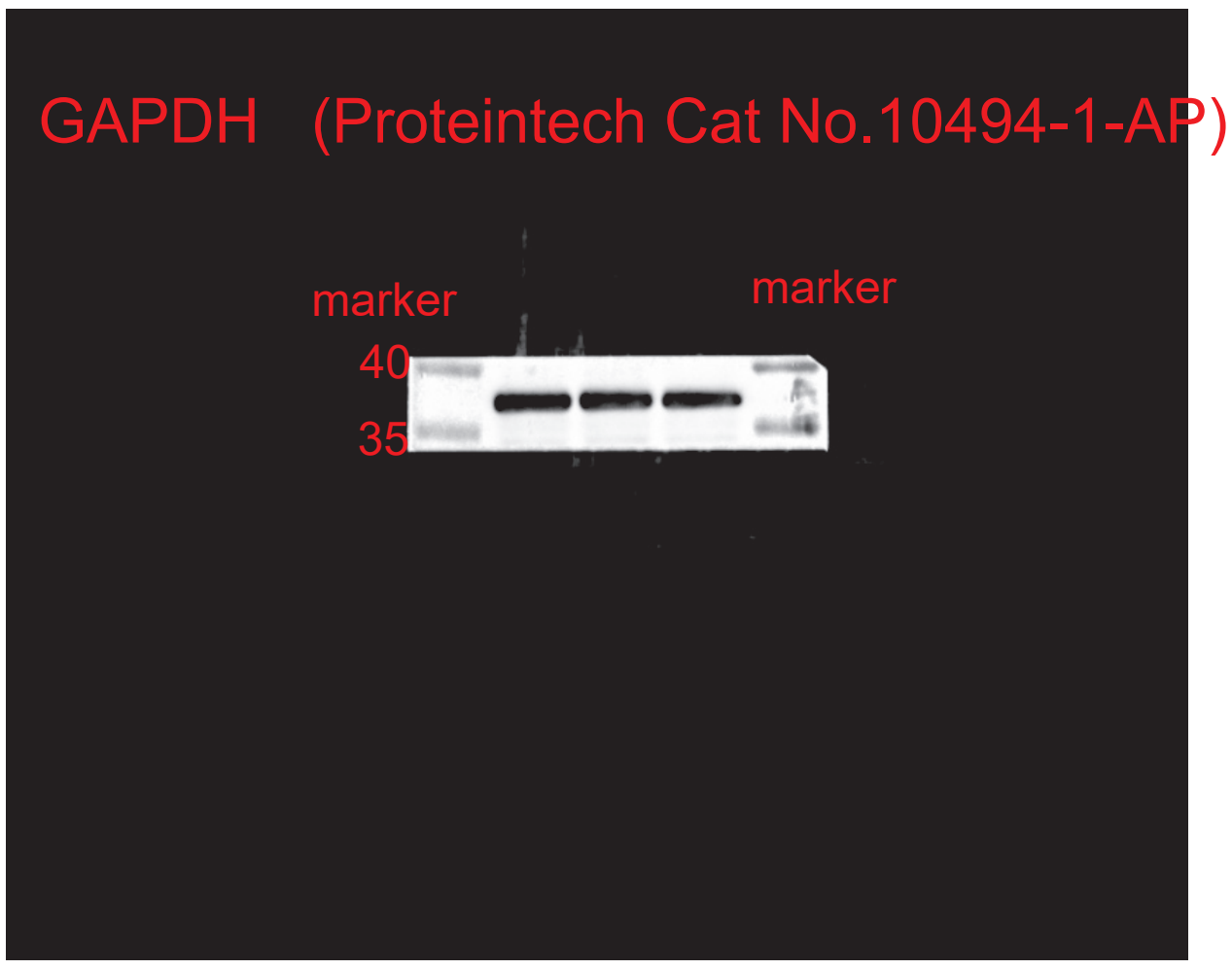
GAPDH (Proteintech Cat No.10494-1-AP)
marker
marker
40
35

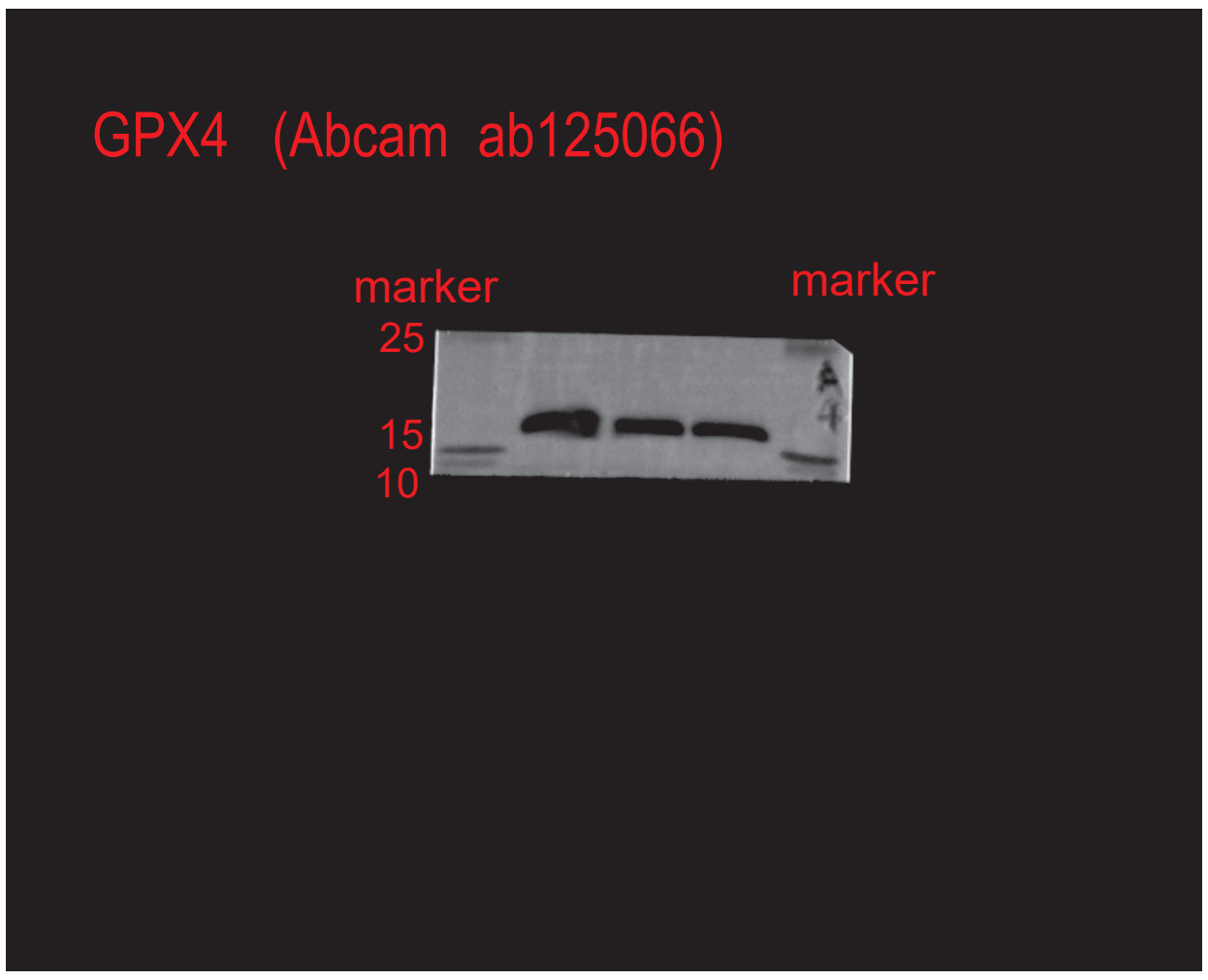
GPX4 (Abcam ab125066)
marker
marker
25
15
10
